# High-protein supplementation in critically ill patients: a systematic review, meta-analysis and umbrella review of existing evidence

**DOI:** 10.3389/fnut.2026.1788894

**Published:** 2026-05-21

**Authors:** Zhiming You, Qike Li, Yuduo Pu, Haoyu Hou, Siyu He, Zihan Yu, Chengxin Xue, Yuan Li, Chengli Wen

**Affiliations:** 1 Department of Critical Care Medicine, The Affiliated Hospital, Southwest Medical University, Luzhou, China; 2 Hejiang County People’s Hospital, Luzhou, China

**Keywords:** critical care patients, critically ill patients, high protein, meta-analysis, umbrella review

## Abstract

**Background:**

Protein serves as a core component of nutritional support for critically ill patients; however, no unified consensus or clinical standard has been established for protein supplementation to date. This gap highlights the urgent need for an updated systematic review and meta-analysis.

**Methods:**

Randomized controlled trials (RCTs) and meta-analyses that met our criteria were included in PubMed, Web of Science, Cochrane Library and Embase English databases up to April 1, 2026. We used meta-analysis, subgroup analysis and umbrella review methods to explore the effects of high-protein and conventional protein supplementation on critically ill patients. We also conducted Trial Sequential Analysis (TSA) to verify the effectiveness of the evidence. The assessment of bias risk was conducted according to the Revised Cochrane’s Risk of Bias tool (ROB 2.0) for RCTs and AMSTAR-2 tool for meta-analysis.

**Result:**

A total of 18 RCTs and 8 meta-analyses are included. In the meta-analysis, no significant association between high-protein supplementation and overall mortality is found (OR 0.99, 95% CI 0.95–1.28, *p* = 0.69; *I^2^* = 0%). In the umbrella review, there are no significant differences in each outcome between high-protein supplementation and conventional protein supplementation. In addition, long term intervention for more than 14 days would slightly increase overall mortality of critically ill patients (OR 1.181, 95% CI 1.002 to 1.391, *p* = 0.047; *I^2^* = 0%).

**Conclusion:**

Compared with high and conventional protein supplementation, high-protein supplementation has no significant impact on clinical outcomes of critically ill patients, and long-term intake might increase overall mortality of patients.

**Systematic review registration:**

Identifier: CRD420250652684.

## Background

Critically ill patients often require prolonged mechanical ventilation and high mortality rates (up to 50% at 1 year), and survivors often suffer from persistent organ dysfunction and severe functional impairment (1). In critically ill patients, stress hormones and inflammatory mediators are activated during the acute phase, leading to increased gluconeogenesis and accelerated muscle proteolysis (2). Critically ill patients suffer from a rapid loss of muscle protein, with a loss of muscle mass up to 18% within the first 10 days of ICU admission, leading to a significant negative nitrogen balance (3), and pronounced muscle atrophy (4). Nutritional support (especially protein supplementation) is considered as a key measurement to improve the prognosis of these patients (5). Studies have suggested that high-protein supplementation may promote protein synthesis, reduce muscle loss, and potentially improve clinical outcomes in critically ill patients (6).

However, studies have shown that both the optimal dose of protein supplementation and the benefits of different doses of protein supplementation in critically ill patients remain controversial (7, 8). According to the EFFORT Protein randomized controlled trial, protein supplementation above 2.2 g/kg/day may have an adverse effect in patients with confirmed acute kidney injury (7). In a population of critically ill patients, increasing the dose of protein supply was not significantly associated with improvements in clinical endpoints or patient-centered outcomes (9). Studies have shown that higher protein supplementation may improve mortality in early-stage patients (10). van Ruijven et al. (11) demonstrated that a high-protein supply of more than 1.2 g/kg appeared to improve changes in nitrogen homeostasis and muscle mass, which may influence short-term and 60-day mortality in critically ill patients. And according to a major randomized controlled trial that was just completed, the findings do not support the intake of enhanced intestinal protein for critically ill patients (12).

Notably, the optimal dose of protein supplementation in critically ill patients remains controversial, with some variation across current guideline recommendations (13, 14). To address this, we updated the evidence base by incorporating the latest published randomized controlled trials (RCTs), thereby substantially enlarging the pooled sample size and enhancing statistical power. We further performed dose-stratified subgroup analyses for high-protein supplementation to identify the optimal protein dosage interval and additionally conducted subgroup analyses across key variables such as intervention duration to explore sources of heterogeneity, generate novel hypotheses, identify potential moderators of treatment response, and inform evidence-based individualized clinical strategies, which represents a key clinical novelty of this study. Methodologically, to our knowledge, this is the first study integrating systematic review, Meta-analysis and umbrella review in this field, and trial sequential analysis (TSA) was also applied to enhance the robustness and reliability of evidence.

Therefore, we performed a systematic review and Meta-analysis, and an umbrella review comparing the effects of high (≥1.2 g/kg/day) and low (<1.2 g/kg/day) protein supplementation in critically ill patients. We analyzed the effects on clinical outcomes including mortality, length of hospital stay, duration of ventilation, and adverse event outcomes, and further conducted in-depth subgroup analyses to comprehensively explore possible sources of heterogeneity as well as effect modifiers. The results of this study may inform clinical practice and future research directions.

## Methodology

### Study design and objectives

The study was conducted in accordance with the Preferred Reporting Items for Systematic Reviews and Meta-Analyses (PRISMA 2020) guidelines (15). It has been registered in the PROSPERO database under the registration number: CRD420250652684. This study aims to compare the effects of high-protein supplementation (≥1.2 g/kg/day) versus conventional protein supplementation (<1.2 g/kg/day) on mortality and other clinical outcomes in critically ill patients.

### Eligibility criteria

We included randomized controlled trials (RCTs) of (1) critically ill patients aged ≥18 years (the patients have been mechanically ventilated for more than 24 h or mortality in the control group must be greater than 5%); (2) protein supplementation equal to or greater than 1.2 g/kg/day is classified as the high-protein supplementation group (experimental group), while less than 1.2 g/kg/day is classified as the conventional protein supplementation group (control group). The difference in protein supplementation between the experimental group and the control group is at least 0.2 g/kg/day. If the dosage is in g/d, it can be converted to g/kg/d using an ideal body weight of 60 kg to satisfy the inclusion criteria. Included studies encompass research on both enteral and parenteral supplementation routes. Amino acid and immune nutrition protocols and studies not meeting the inclusion criteria were excluded; (3) studies reporting overall mortality were included.

We included the following meta-analyses: (1) Adult (≥18 years old) critically ill patients; (2) Comparing high-protein supplementation with conventional protein supplementation (allowing the threshold defined in the original text or not specifying the threshold but comparing conventional protein, there is no requirement for the difference between protein provision groups); (3) The primary outcome is overall mortality; secondary outcomes included other mortality other than overall mortality, length of hospital stay, length of intensive care unit (ICU) stay, duration of mechanical ventilation, and adverse events (at least one of the above outcomes was reported in the included meta-analyses).

### Exclusion criteria

Literature that did not match the study types, focused on amino acids and immune nutrition, included critically ill patients under the age of 18 or non-critically ill patients, or did not provide necessary data or information was excluded.

### Information source and search strategy

Literature was searched in four English databases: PubMed, Web of Science, Cochrane Library, and Embase. The search strategy was conducted based on the framework of patients, intervention, comparison, outcomes, and study design (PICOS), as shown in Supplementary File A Table 1. The search was carried out in two stages: stage 1 focused on retrieving RCTs, and stage 2 focused on retrieving meta-analyses. The search deadline was April 1, 2026. Details on the search strategy for the meta-analyses can be found in Supplementary File A Tables 2–5, and details for umbrella review can be found in Supplementary File A Tables 6–9.

### Study selection

**Figure 1:**
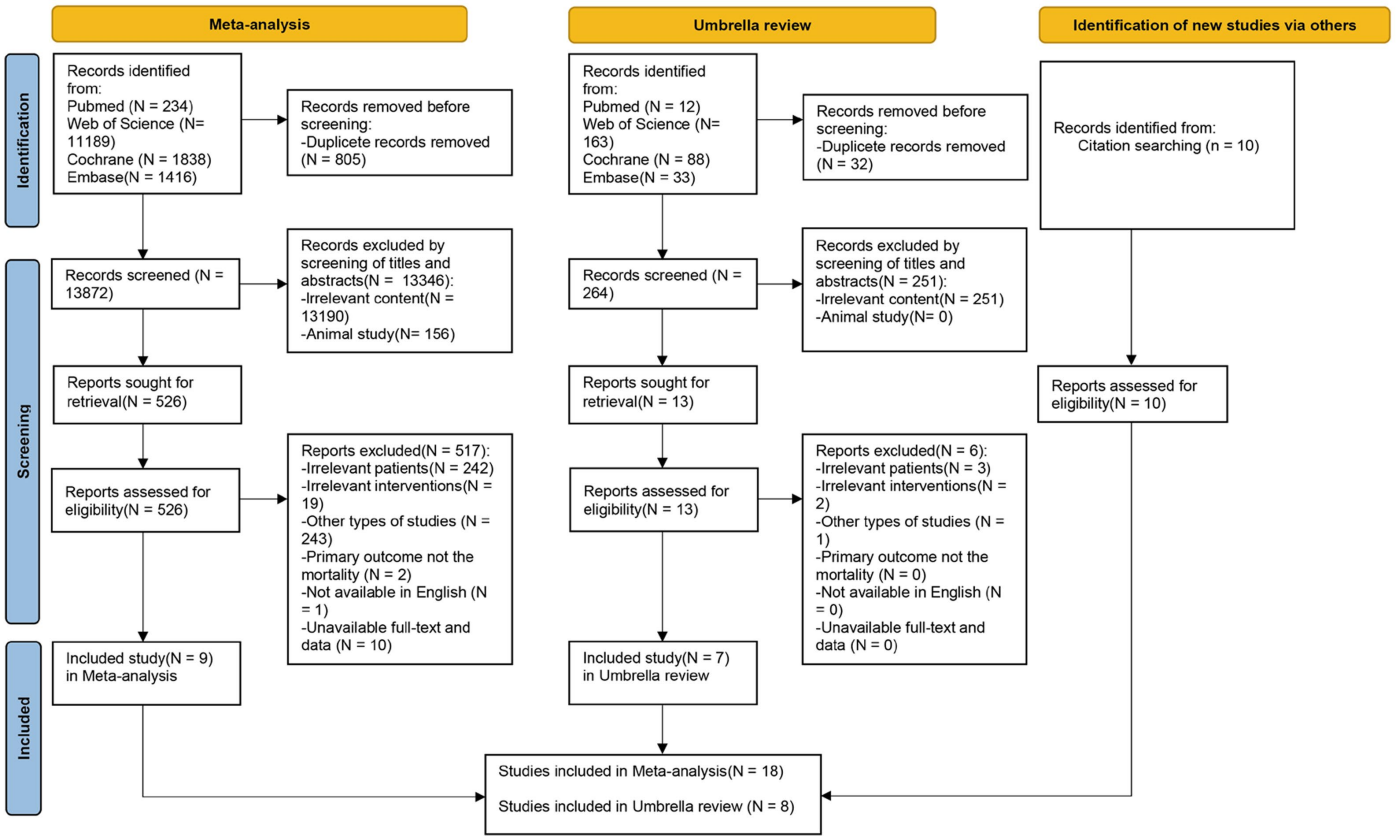
PRISMA flow diagram for updated meta-analysis and systematic reviews, and umbrella review.

Two independent authors (Zhiming You and Qike Li) screened the literature by reading the titles and abstracts. Studies that are uncertain were determined by thoroughly reading the full text to decide if they should be included. Inter-rater agreement for full-text screening was assessed using Cohen‘s kappa (*κ*) statistic in SPSS Statistics 26 (*κ* > 0.80 indicating almost perfect agreement) (16). If there are still disagreements, a third author (Yuduo Pu) would be consulted. Any studies that did not meet the PICOS inclusion criteria were excluded. The reason for exclusion should be clearly recorded. Other eligible studies that were not included were manually added. The screening process for the included literature is displayed in PRISMA flow diagram (Figure 1).

### Data extraction

The data collected from RCTs included title, author, publication year, country, study center, study year range, sample size of experimental and control groups, gender, Body Mass Index (BMI), age, Acute Physiology and Chronic Health Evaluation II (APACHE II), Sequential Organ Failure Assessment (SOFA) score, Simplified Acute Physiology Score II (SAPS II), nutritional strategy, duration, protein dosage (g/kg/d), and data on clinical outcomes.

The data collected from meta-analysis studies included publication details (author, publication year, end of search, and country), original research type, N (the number of studies included in), n (the number of patients included in), patients, gender, age, protein dosage, outcomes, conflict of interest, and data on clinical outcomes.

We used the graphic digitization function of Origin64 in data extraction that data extraction was performed by two reviewers (Zhiming You and Qike Li). In case of any discrepancies in the extracted values, a third author (Yuduo Pu) was consulted for confirmation.

### Risk of bias assessment

The assessment of risk of bias was conducted according to the Revised Cochrane’s Risk of Bias tool (ROB 2.0) for RCTs (17), and A Measure Tool to Assess Systematic Reviews-2 (AMSTAR-2) tool for meta-analysis (18).

The risk of bias in RCTs was assessed based on the randomization process, blinding, completeness of outcome data, and selective reporting of the included studies. AMSTAR 2 has 10 original domains and 16 items in total (18).

Quality assessments will be performed by two independent authors, and the results of the RCTs will be rated as “high risk,” “some concerns,” or “unclear” risk of bias (17). Overall confidence in the results of the review was classified as high (No or one non-critical weakness), moderate (More than one non-critical weakness), low (One critical flaw with or without non-critical weaknesses), or critically low (More than one critical flaw with or without non-critical weaknesses) (18). In case of disagreement, we consulted another author.

### Outcomes

*Meta-analysis*: The primary outcome is overall mortality. If overall mortality is not reported, 28-day, 90-day, ICU, or hospital mortality was used as a substitute. Secondary outcome measures include other mortality (28-day mortality, 60-day mortality, hospital mortality, and ICU mortality), length of ICU stay, length of hospital stay, mechanical ventilation time, and adverse event rate (diarrhea rate, pneumonia infection rate, RRT (renal replacement therapy) rate, insulin requirement).

*Umbrella review*: The primary outcome is overall mortality. Secondary outcomes include other mortality excluding overall mortality, length of hospital stay, length of ICU stay, mechanical ventilation time, and adverse events (at least one of the above outcomes was reported in the included meta-analyses).

### Subgroup analysis

Subgroup analysis is used to explore the variables that affect the effect size and investigate causes of heterogeneity, based on critical illness severity (evaluated by SOFA, APACHE II, and SAPS II scores), nutritional strategy [enteral nutrition (EN) alone or a combination of enteral and parenteral nutrition (PN)], study year range (taking 2020 as the boundary; if the study year includes 2020, it is regarded as after 2020), intervention duration(short and medium-term intervention(≤14 days) and long term intervention (>14 days)), study center. We conducted a subgroup (literature was divided into subgroups of 1.2–1.5 g/kg/day, 1.5–1.8 g/kg/day, 1.8–2.0 g/kg/day, and ≥2.0 g/kg/day) analysis of the high-protein supplementation group to find a safe and effective dosage range.

The classification of intervention duration is based on clinical practices and previous research, where 14 days is a commonly accepted threshold for defining intervention timelines (19, 20). According to the research results of Knaus et al. (21), the APACHE II score was strongly positively correlated with in-hospital mortality, and mortality significantly increased when the score was ≥25. Therefore, we adopted an APACHE II score of ≥25 points as the criterion for defining high-risk critically ill patients, and a score of <25 points was defined as low and medium-risk patients for subgroup analysis. According to the prospective study of Vincent et al. (22), the SOFA score was significantly correlated with mortality in critically ill patients, and mortality entered a rapid increase range when the total score was ≥6. Our study referred to this trend and defined patients with a SOFA score of ≥6 as high-risk critically ill patients, and a score of <6 points was defined as low and medium-risk patients. The patients were divided into two subgroups according to the SAPS II score: low and medium-risk patients (SAPS II < 30) and high-risk patients (SAPS II ≥ 30). The selection of this cut-off point referred to the study of J. R. Le Gall et al., which confirmed that, in patients in the transitional intensive care unit, SAPS II could effectively predict in-hospital mortality, and a score of around 30 roughly corresponded to the patient group with a significantly increased risk of death (23). APACHE II is more commonly used and should be given priority. SOFA is the second most used, and if the article does not mention APACHE II but includes SOFA, it is reasonable to consider SOFA as the judgment criteria. Finally, if neither APACHE II nor SOFA is mentioned in the article, the choice falls to SAPS II.

### Sensitivity analysis

To assess the robustness of pooled effect estimates and minimize potential bias arising from data processing and extraction limitations, sensitivity analyses were performed by excluding three specific categories of studies. Specifically, studies requiring 60 kg weight conversion were excluded as standardized weight conversion of this type may introduce systematic errors and deviate from the actual baseline characteristics of patients (24–27), which can lead to biased estimations of protein dose effects. Studies with imputed or assumed actual protein intake were also excluded because such data are unable to reflect the real status of protein exposure (28), which may lower the accuracy and authenticity of core intervention data. In addition, studies for which protein dose was derived from graphical figures were excluded since data digitized from graphical figures may retain potential numerical uncertainty and reduced precision (25, 29), which can introduce bias into the key intervention dose. These sensitivity analyses were specifically conducted for the primary outcome of overall mortality, the long-term intervention subgroup of overall mortality, as well as secondary outcomes including length of hospital stay, length of ICU stay, and mechanical ventilation time. For the remaining outcomes and subgroup analyses, the number of eligible included studies was relatively small, and further exclusion of the three categories of studies would lead to an excessively small pooled sample size, increase the risk of type I and type II statistical errors, and diminish the statistical power and clinical reference value of the analytical results. Therefore, sensitivity analyses were not performed for these indicators and subgroups.

Additionally, an exploratory supplementary sensitivity analysis was performed for the primary outcome of overall mortality, which was restricted to studies that directly reported definitive overall mortality instead of surrogate endpoints including 28-day, 90-day, ICU and hospital mortality. Only 4 included studies were eligible for this supplementary analysis. Given the extremely limited number of included studies, this analysis was conducted in an exploratory manner to verify the directional consistency of the primary findings, rather than to perform formal confirmatory statistical inference.

### Statistical analysis

The meta-analysis was conducted by RevMan (Version 5.4) and Stata (Version 18.0). Due to clinical and methodological heterogeneity across the included studies, a random-effects model was chosen. Mean difference (MD) with 95% confidence interval (CI) was employed for continuous data, and odds ratio (OR) with 95% CI was employed for dichotomous data. Heterogeneity was evaluated via the Q-test (*p* < 0.1 and I^2^ > 50% indicate substantial heterogeneity, while *p* > 0.1 and *I^2^* < 50% indicate no significant heterogeneity). *p* < 0.05 was considered statistically significant. The umbrella review was conducted by Stata (Version 18.0). Mean difference (MD) with 95% confidence interval (CI) was employed for continuous data, and relative risk (RR) with 95% CI was employed for dichotomous data. For the meta-analyses included in the umbrella review, we calculated the degree of overlap between reports. Corrected coverage areas (CCA) of 5, 6–10%, 11–15%, and >15% corresponded to slight, moderate, high, and very high overlaps, respectively (30).

### Potential publication bias

A funnel plot was used to display potential publication bias, and the Egger test and Begg test were used to assess publication bias by Stata (version 18.0).

### Trial sequential analysis (TSA)

TSA is a new statistical method that introduces the concept of sequential analysis into clinical research and then extends it to meta-analysis, which can better control Type I errors (rejecting a true null hypothesis) and Type II errors (failing to reject a false null hypothesis). It provides researchers with a reference by calculating the required information size (RIS)—the total sample size needed for meta-analysis to reach reliable conclusions—as well as the thresholds for hypothesis testing and the futility boundary. When the total sample size included in a meta-analysis reaches the RIS, or the Z-curve of hypothesis testing intersects with the provided thresholds or futility line, it indicates that the statistical test results of the analysis tend to stabilize. This analysis was conducted by TSA software (version 0.9.5.10 Beta; Copenhagen Trial Unit, Center for Clinical Intervention Research, Rigshospitalet, Copenhagen, Denmark) (31).

### Certainty of the evidence

The Grading of Recommendations Assessment, Development and Evaluation (GRADE) approach was used to rate the certainty of the evidence (32). The level of certainty of the evidence was categorized as high, moderate, low, or very low (33). GRADE profiler (version 3.6.1) was used.

## Results

### Study selection

In the first stage, we retrieved a total of 14,677 literature (234 from PubMed, 11,189 from Web of Science, 1,838 from Cochrane Library, and 1,416 from Embase). In the second stage, we retrieved a total of 296 (12 from PubMed, 163 from Web of Science, 88 from Cochrane Library, and 33 from Embase). Finally, we included 18 RCTs (6–8, 24–29, 34–42) and 8 meta-analyses (9–11, 43–47). The PRISMA flow diagram of the literature screening is shown on Figure 1.

### Characteristics and original outcome data of the included studies

The characteristics of the 18 included RCTs were summarized in Supplementary File A Table 10. The period of these researches ranges from 2009 to 2023. Among the included RCTs literature, 6 only used enteral nutrition (8, 26–28, 36, 42), while the remaining 12 gave priority to using enteral nutrition and combined with parenteral nutrition when necessary (6, 7, 24, 25, 29, 34, 35, 37–41). Intervention time ranged from 3 days to 90 days. 9 had an intervention time of less than or equal to 14 days (25–27, 29, 34–36, 40, 42), and 9 had an intervention time of more than 14 days (6–8, 24, 28, 37–39, 41). According to the defined severity of critically ill patients, patients in 12 studies were classified as low and medium-risk patients (6–8, 25–27, 29, 37, 39–42), and patients in 6 studies were classified as high risk patients (24, 28, 34–36, 38). The average age of the included patients ranged from 48.31 y (years) to 69.24 y, and the proportion of male gender ranged from 40.9 to 95%. The average BMI of the participants ranged from 21.3 to 30.7 kg/m^2^.

The number of studies included in the 8 meta-analyses ranges from 4 to 29, and sample sizes range from 1730 to 7,190. These meta-analyses were published from 2017 to 2025. The average age of the participants ranged from 32.7 to 74.2(year). The proportion of male participants ranged from 37.1 to 100%. Most literature included in meta-analyses is RCTs. The included outcomes are overall mortality, hospital mortality, ICU mortality, 28-day mortality, mechanical ventilation time, length of ICU stay, length of hospital stay, and infectious complications. The information including the definitions of included patients, the types of diseases, the conclusions, and conflicts of interest can be found in Supplementary File Tables 11, 12. In the study by Kagan I (27), there were three groups. The first group received conventional physical rehabilitation therapy. The latter two groups underwent Cycle Ergometry (CE), combined with either high-protein or the conventional protein supplementation diets. The only variable among these two groups was the protein dosage. We adopted the data from the latter two groups, which combined CE with different protein intakes.

The original outcome data of included RCTs are presented in Supplementary File A Tables 13, 14, and the original outcome data of the included meta-analyses are presented in Supplementary File A Tables 15, 16. Inter-rater agreement for full-text screening, assessed using Cohen’s kappa, was excellent for both the meta-analysis (Supplementary File B4 Figure 15a, *κ* = 0.940) and the umbrella review (Supplementary File B4 Figure 15b, *κ* = 0.847).

### Protein dosage

The protein dose unit of some RCTs is g/d (24–26). We converted the ideal body weight of 60 kg to g/kg/d to participate in the grouping of protein dose subgroups. In addition, in one study (28), the conventional protein supplementation group do not specify the actual protein amount, but its target protein was 1.2 g/kg/d. Considering that the actual protein amount is usually less than the target protein amount in experimental studies, the protein amount in this study was less than 1.2 g/kg/d, which met the inclusion criteria.

### Data extraction, transformation and integration

In one of included RCTs, the authors only provided images of protein dose (25). In the other RCT, the authors only provided images of 28-day mortality, length of ICU stay, length of hospital stay, and mechanical ventilation duration outcomes (29). Therefore, we used the graphic digitization function in Origin64 to extract these data (Supplementary File B3 Figures 12a-f). Since the data collection standards for each RCT experiment are different, in some literature, the continuous variable data are quartiles (lower quartile, median, upper quartile), but RevMan 5.4 data analysis and processing require the mean and standard deviation. So we used a website that could convert quartiles into mean and standard deviation (48). The website is based on these two studies (49, 50). According to the prompts of the website, the biased data that cannot be converted are excluded. Specifically, the quartile-formatted data of mechanical ventilation time from Danielis (25), as well as length of hospital stay and length of ICU stay from W. Xiong (29), were converted into mean and standard deviation using the aforementioned conversion website, ensuring compatibility with the data requirements of RevMan 5.4 for subsequent statistical analysis. In the study conducted by Bukhari et al. (26), they were divided into the conventional protein intake group, high-protein polymeric group, and the oligomeric group. Each of these three groups was further divided into patients with traumatic brain injury (TBI) and patients without traumatic brain injury, that is, a total of 6 groups. Since we did not limit the patient types, the protein dosage ranges of the high-protein polymeric and oligomeric groups all belonged to high-protein dosage. Moreover, to avoid sample size fragmentation, improving the sample size and the efficiency of statistical tests, the continuous variables of the two groups of TBI patients and non-TBI patients in the conventional protein group were combined with the mean and standard deviation (SD). Similarly, the two types of patients included in each of the high-protein polymeric and oligomeric groups were combined, totaling 4 groups. The mean and standard deviation (SD) were also combined (49). We designed two codes for calculation using Python 3.10 based on the formulas of combined mean and combined standard deviation (SD). The principles of the formulas and the codes can be found in Supplementary File C1, 2.

Among the included meta-analysis, in the study of Davies et al. (43), overall mortality adopted odd ratio (OR), but most other studies using relative risk (RR), so we converted OR to RR based on Anthony’s research (51).

### Risk of bias assessment

For the ROB 2.0 assessment, among the 18 RCTs, there is 1 high-risk study (26), and 4 low-risk studies (6, 7, 25, 39), and other studies are classified as medium-risk bias studies (Supplementary File B1 Figure 1). The AMSTAR-2 assessment show that 3 (9, 44, 45), and 2 (43, 46), 2 (11, 47), and 1 (10) meta-analysis had high, moderate, low, and critically low quality, respectively (Supplementary File A Table 17).

### Meta-analysis

#### Mortality

**Figure 2:**
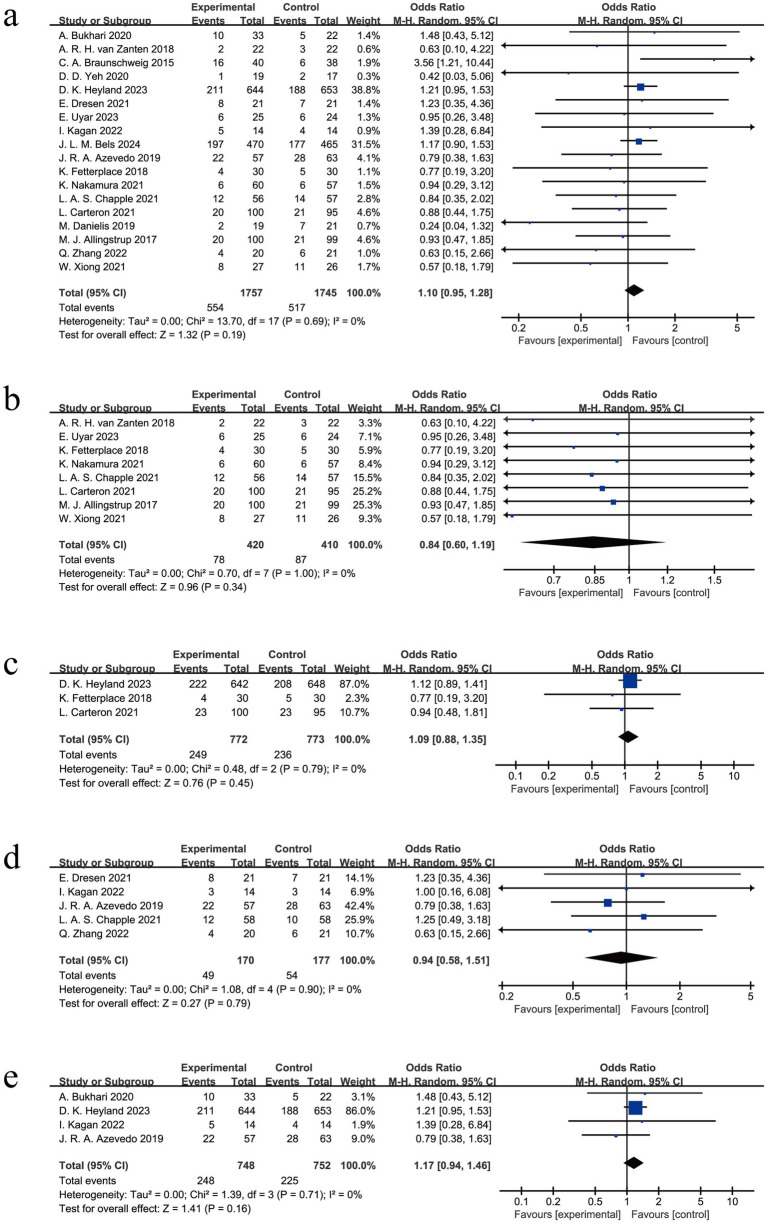
Meta-analysis of mortality outcomes: **(a)** Overall mortality, **(b)** 28-day mortality, **(c)** 60-day mortality, **(d)** ICU mortality, **(e)** hospital mortality.

All the included studies reported mortality outcome (*n* = 3,502) (6–8, 24–29, 34–42). For the primary outcome, overall mortality, there was no significant difference between the high-protein group and the conventional protein group (Figure 2a, OR 0.99, 95% CI 0.95 to 1.28, *p* = 0.19; I^2^ = 0%) (6–8, 24–29, 34–42). As for the secondary outcomes including 28-day mortality (28, 29, 34, 36, 37, 39–41), 60-day mortality (7, 36, 39), ICU mortality (8, 27, 35, 37, 38), and hospital mortality (7, 26, 27, 35), there were also no significant differences between the high-protein group and the conventional protein group either (Figures 2b–e).

**Figure 3:**
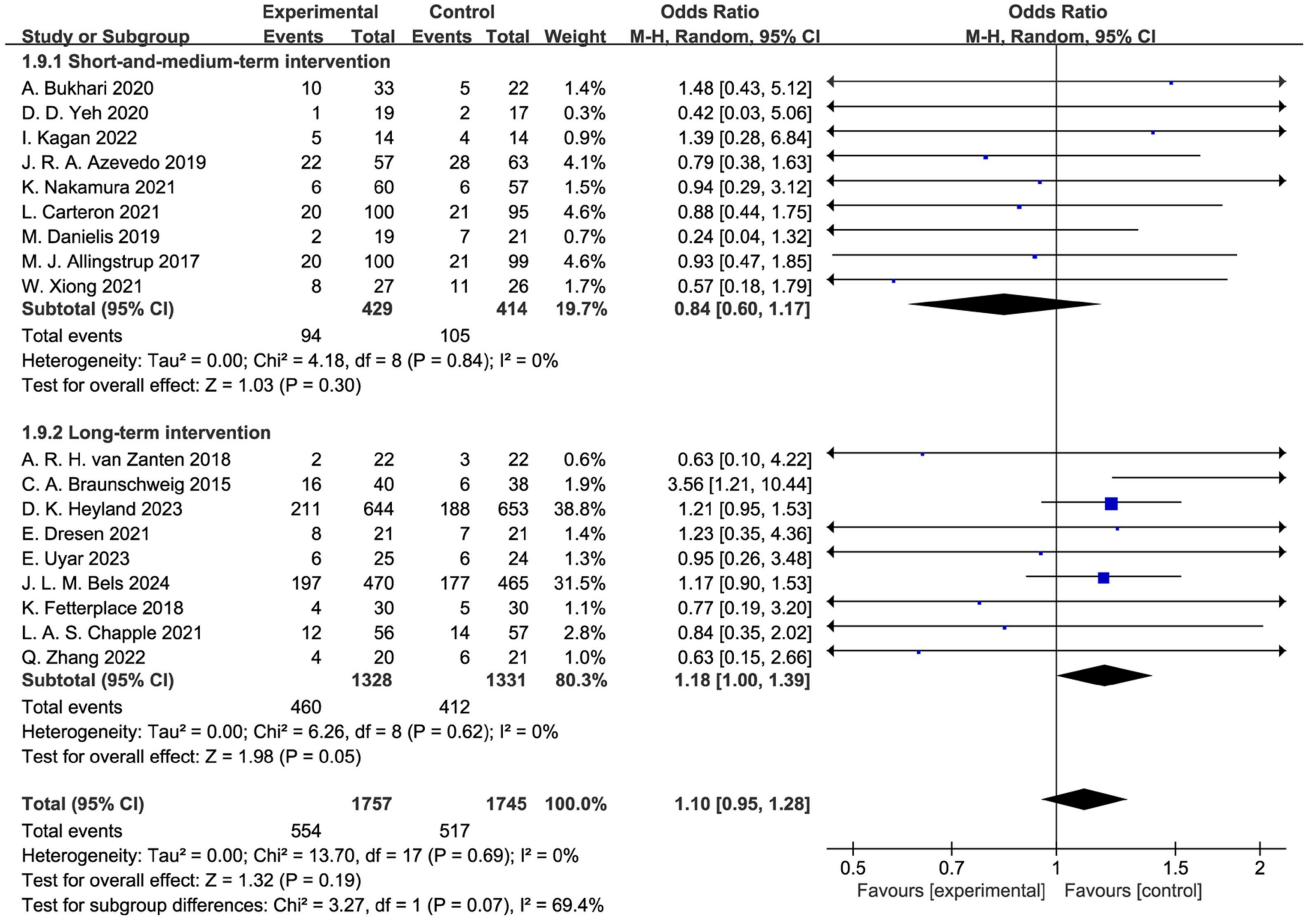
Overall mortality (subgroup analysis of short and medium-term intervention vs. long term intervention).

Among the 6 subgroup analyses of overall mortality, short and medium-term intervention/long term intervention subgroup found that long term intervention of high-protein lasting more than 14 days might increase overall mortality of critically ill patients (Figure 3, OR 1.18, 95% CI 1.00 to 1.39, *p* = 0.05; *I^2^* = 0%; test for subgroup differences *p* = 0.07) (6–8, 24, 28, 37–39, 41), and no significant effect was found in the short and medium-term subgroup (Figure 3, OR 0.84, 95% CI 0.60 to 1.17, *p* = 0.30; *I^2^* = 0%; test for subgroup differences *p* = 0.07) (25–27, 29, 34–36, 40, 42). Since the lower limit of the 95% confidence interval of the result through RevMan 5.4 was exactly 1, we conducted a more precise calculation using Stata 18.0. The result was OR 1.181, 95% CI 1.002 to 1.391, *p* = 0.047; *I^2^* = 0% (Supplementary File A Table 18), supporting the conclusion that long term intervention of high-protein would slightly increase overall mortality of critically ill patients. However, due to the non-significant interaction test for subgroup differences (*p* = 0.07), this between-subgroup difference should be interpreted with caution. No significant differences were observed in the analyses of the other five subgroups (Supplementary File B1 Figures 2a, c–f).

#### Secondary outcomes

For length of hospital stay (Supplementary File B1 Figure 3a, MD 0.65, 95% CI –2.78 to 4.07, *p* = 0.71; *I^2^* = 37%) (24, 26, 27, 29, 34, 37, 39, 41), length of ICU stay (Supplementary File B1 Figure 3b, MD 0.16, 95% CI –1.50 to 1.82, *p* = 0.85; *I^2^* = 41%) (8, 24, 26, 27, 29, 34–39, 41), mechanical ventilation time (Supplementary File B1 Figure 3c, MD –0.55, 95% CI –1.44 to 0.34, *p* = 0.23; *I^2^* = 0%) (24, 25, 27, 29, 35, 36, 38–41), there were no significant differences in these outcomes.

**Figure 4:**
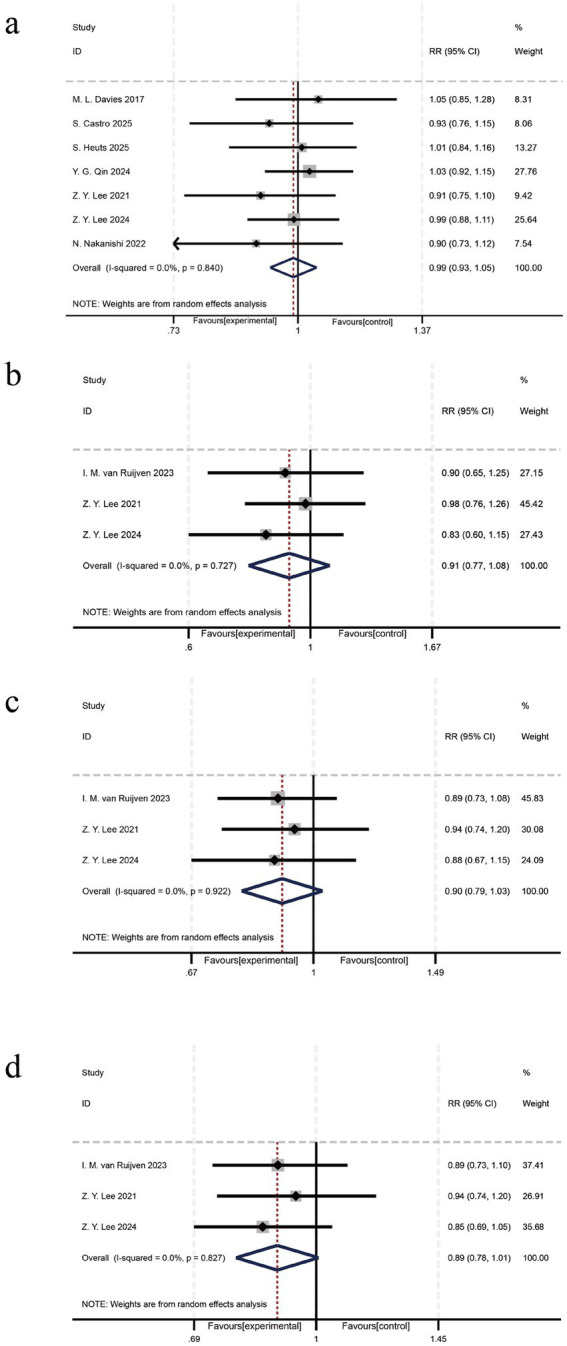
Mortality outcomes of the umbrella review **(a)** Overall mortality, **(b)** 28-day mortality, **(c)** Hospital mortality, **(d)** ICU mortality.

In the short and medium-term intervention/long term intervention subgroup of length of hospital stay, we found that under the short and medium-term intervention premise of less than 14 days, length of hospital stay in the high-protein group was shorter than that in the conventional protein group (26, 27, 29, 34). And there was a significant difference (Supplementary File B2 Figure 4B, MD –2.67, 95% CI –5.09 to −0.25, *p* = 0.03; *I^2^* = 0%; test for subgroup differences *p* = 0.02), and no significant effect was found in the long term intervention subgroup (Supplementary File B2 Figure 4B, MD 3.30, 95% CI –1.07 to 7.67, *p* = 0.03; *I^2^* = 0%; test for subgroup differences *p* = 0.02) (24, 37, 39, 41). However, there was no statistical difference in other five subgroups (Supplementary File B2 Figures 4A, C–F). In the subgroup of low and medium-risk/high risk patients with length of ICU stay, we found that the length of ICU stay of low and medium-risk patients who received high-protein supplementation was shorter (Supplementary File B2 Figure 5a, MD –1.28, 95% CI –2.46 to −0.10, *p* = 0.03; *I^2^* = 0%; test for subgroup differences *p* = 0.20) (8, 26, 27, 29, 37, 39, 41), and no significant effect was found in the high risk patients subgroup (Supplementary File B2 Figure 5a, MD 0.98, 95% CI –2.27 to 4.23, *p* = 0.55; *I^2^* = 0%; test for subgroup differences *p* = 0.20) (24, 34–36, 38). However, due to the non-significant interaction test for subgroup differences (*p* = 0.20), this between-subgroup difference should be interpreted with caution. And there was no statistical difference in other subgroups (Supplementary File B2 Figures 5b-f). There was no statistical significance in the 6 subgroups of mechanical ventilation time (Supplementary File B2 Figures 6a-f).

#### Adverse event outcomes

There were no significant differences in adverse event including diarrhea rate (36, 39–41), pneumonia infection rate (6, 34, 36, 38, 40), insulin requirement (7, 24, 40), and RRT rate (34, 37, 38) (Supplementary File B2 Figures 7a-d).

### Umbrella review

#### Mortality

There were no significant differences in the outcomes of overall mortality (9, 10, 43–47), 28-day mortality (9, 11, 45), hospital mortality (9, 11, 45), and ICU mortality (9, 11, 45) in umbrella review (Figures 4a–d).

#### Other outcomes

There were no significant differences in the outcomes of mechanical ventilation time (9, 11, 43–46), length of hospital stay (9, 11, 43, 45–47), length of ICU stay (9, 11, 43, 45, 46), and infectious complication rate (9, 11, 44, 45) (Supplementary File B2 Figures 8a–d).

### Sensitivity analysis

Among the five outcomes included in the sensitivity analyses, the long-term intervention subgroup of overall mortality was the only one with altered findings. Its initially positive result turned nonsignificant after excluding the three categories of studies (Supplementary File B4 Figure 13b, OR 1.15, 95% CI 0.98 to 1.36, *p* = 0.09; *I^2^* = 0%), suggesting that this outcome was not robust. For the remaining four outcomes, all of which showed no significant between-group differences in the primary meta-analysis, the pooled results remained consistent and nonsignificant following sensitivity analysis: overall mortality (Supplementary File B4 Figure 13a, OR 1.10, 95% CI 0.94 to 1.28, *p* = 0.22; *I^2^* = 0%), length of hospital stay (Supplementary File B4 Figure 13c, MD 1.57, 95% CI –3.49 to 6.63, *p* = 0.54; *I^2^* = 31%), length of ICU stay (Supplementary File B4 Figure 13d, MD 0.93, 95% CI –1.08 to 2.95, *p* = 0.36; *I^2^* = 31%), and mechanical ventilation time (Supplementary File B4 Figure 13e, MD –0.14, 95% CI –1.31 to 1.04, *p* = 0.82; *I^2^* = 21%).

Furthermore, an exploratory sensitivity analysis restricted to studies directly reporting definitive overall mortality was performed for the primary outcome. The pooled result remained nonsignificant without between-group differences (Supplementary File B4 Figure 14, OR 1.08, 95% CI 0.42 to 2.76, *p* = 0.88; *I^2^* = 63%), which was directionally consistent with the primary meta-analysis. The elevated heterogeneity observed in this analysis was attributable to the extremely small number of included studies.

### Trial sequential analysis (TSA)

The TSA results were summarized in Supplementary File B3 Figures 9a–d, indicating that the current systematic review and meta-analysis did not achieve the required information sizes to detect the pre-specified effect sizes for overall mortality, the long term intervention subgroup analysis of overall mortality, the short and medium-term intervention subgroup analysis of length of hospital stay, and low and medium-risk patients with length of ICU stay and showing that more trials are needed to draw clear conclusions on these results.

### Publication bias

The funnel plot of overall mortality as the primary outcome did not show evidence of asymmetry, and neither did the Egger and Begg test results in Stata18.0 (Supplementary File B3 Figure 10). Therefore, no clear evidence of publication bias was detected in included studies.

### GRADE certainty assessments

The conclusion that higher protein supplementation does not affect overall mortality of critically ill patients is moderately certain evidence, and other outcomes are also moderately certain evidence (Supplementary File B3 Figure 11).

### Corrected covered area (CCA)

The Corrected Covered Area of the primary outcome, overall mortality, was 29.50%, indicating a very high degree of overlap (Supplementary File A Table 19).

## Discussion

This study, through systematic review and meta-analysis, explored the effect of high-protein supplementation on critically ill patients. The results showed that high-protein supplementation did not significantly improve the clinical outcomes of critically ill patients, including overall mortality, length of ICU stay, and mechanical ventilation time. Furthermore, in combination with the current clinical guidelines regarding the recommended protein dosage, there is no difference in the clinical outcomes of critically ill patients between high-protein supplementation and low-protein supplementation. However, based on the widespread clinical consensus on an intervention period of 14 days, we explanatively set 14 days as the demarcation line for our subgroups (13, 52). And according to a recent larger randomized controlled trial, the study showed no support for the intake of enhanced intestinal protein in critically ill patients (12). It is worth noting that in this study, the “protein dosage” was based on the reported supply levels, without distinguishing between prescriptions and actual intake, and was analyzed in a combined manner. This was mainly because most studies did not report actual intake or attainment of the target, which limited the stratified analysis. In the ICU setting, actual intake is often lower than the prescription target, which may affect the interpretation of the results. Therefore, the results of this study should be understood as a comparison of protein supply strategies rather than differences in actual exposure and should be interpreted with caution and further verified.

This study revealed that within a shorter intervention period (≤14 days), there was a certain correlation between the high-protein group and a shorter hospital stay (Supplementary File B2 Figure 4B). However, this result should be interpreted with caution as the current evidence is insufficient to support a definite causal relationship. More high-quality research will be needed in the future to verify this. This might be related to the fact that short-term (≤14 days) high-protein supplementation can help the body in acute stage restore normal physiological functions as soon as possible. Firstly, adequate protein intake in the early stage (1.2–2.0 g/kg/day) can quickly correct the negative nitrogen balance, promote the positive nitrogen balance, alleviate the catabolism of the body in the acute stage, and provide a crucial guarantee for maintaining the functions of important organs and the immune defense system (53). Multiple studies have shown that this intervention can also promote the recovery of human respiratory muscle strength, accelerate the withdrawal from mechanical ventilation, and reduce the incidence of ICU-acquired myasthenia (7, 54). Secondly, high-protein nutritional support reduces the risk of intestinal bacterial translocation by maintaining the normal function of the intestinal barrier, thereby reducing the occurrence of secondary infections and related complications (55). It is notable that early protein supplementation can also promote patients to enter the recovery stage more quickly by regulating the inflammatory response (reducing IL-6 levels) and optimizing the mechanism of glucose metabolism in the body (56). These combined effects ultimately played a crucial role in helping patients significantly shorten their hospital stay. Although we propose the ‘time window’ hypothesis to interpret the stratified results by intervention duration (≤14 days vs. >14 days), it is critical to acknowledge that this hypothesis lacks direct validation in our study. Since the 14-day threshold has certain clinical practice basis but the subgroup results based on this demarcation point still need to be interpreted with caution. Especially, the TSA results show that some key outcomes have not yet reached the required information volume, thereby limiting the robustness of these results. Therefore, these findings should be regarded as exploratory results and still require more high-quality studies for verification. Key elements required to test time-dependent effects were not assessed. Thus, our support for this hypothesis must be regarded as conjectural, not as evidence-based confirmation, and its validity awaits future dedicated investigations. Our research indicates that for patients with low and intermediate critical conditions (classified based on the SOFA score and other critical condition scores), compared with the conventional protein supplementation group, the high-protein supplementation group can reduce the length of ICU stay for critically ill patients. Which is consistent with previous studies. The reason might be the effect of multiple synergistic mechanisms. Due to the relatively well-preserved metabolic regulatory ability of such patients, Therefore, it can more effectively utilize proteins and exogenous amino acids for the synthesis of important substances, thereby rapidly improving respiratory muscle function and enhancing exercise endurance. Studies have shown that targeted protein supplementation can also improve the nitrogen balance status of such patients, helping them achieve positive nitrogen balance 2–3 days earlier than conventional doses and directly promoting the recovery of early activity capacity of the body (46). Meanwhile, moderate protein load does not increase the burden on organ functions. Instead, it reduces the secondary infection rate by approximately 30% by maintaining the integrity of the intestinal mucosa (increasing the expression of tight junction proteins) and regulating the Th1/Th2 immune balance (55, 57). The risk stratification of critically ill patients using predefined thresholds for APACHE II, SOFA, and SAPS II scores should be interpreted as exploratory. Although these thresholds were selected based on prior literature, they were not prospectively defined in our protocol, and the subgroup analyses lacked dedicated statistical power to detect interactions. Thus, these findings serve primarily to generate hypotheses regarding differential treatment effects across risk strata, rather than to confirm clinically actionable thresholds. In addition, we conducted a sensitivity analysis on the main outcomes, including overall mortality, the subgroup of overall mortality stratified by intervention time, length of hospital stay, length of ICU stay, and mechanical ventilation time. The results showed that except for the subgroups of overall mortality stratified by intervention time, no significant differences were observed between the experimental group and the control group in the other outcomes under different analysis conditions, suggesting that the overall results are relatively robust. However, the results of the intervention time-related subgroups showed certain instability in the sensitivity analysis, further supporting the cautious interpretation of these results and emphasizing the need for more research to verify them. Furthermore, an exploratory sensitivity analysis restricted to studies directly reporting overall mortality yielded consistent, non-significant results compared with the primary analysis, despite increased heterogeneity attributable to the small number of included studies. These findings further support the robustness of the primary conclusions regarding overall mortality. Meanwhile, we fully recognize that the subgroup analysis is inherently exploratory. Therefore, we emphasize that these results should be regarded as preliminary findings that generate hypotheses and are only for reference in subsequent research.

During a longer clinical intervention period (>14 days), the high-protein supplementation group slightly increased the mortality rate of critically ill patients compared with the conventional protein supplementation group (7, 36). However, the TSA results of this subgroup analysis may be affected by random errors or limitations in sample size. The current evidence is not sufficient to support a definite association between the two, and it should be interpreted with caution and further research is needed for verification. This phenomenon may be related to the potential negative impact of long-term high-protein intake on the metabolism and organ function of critically ill patients. Because during the long-term intervention period (>14 days), patients are often in a state of high metabolism and high decomposition. At this time, excessive protein intake may exceed the metabolic capacity of the liver and kidneys of the body, increasing the risk of azotemia. And it may aggravate the accumulation of metabolic wastes like toxins, thereby further damaging renal function and even leading to uremia (58). In addition, long-term high-protein intake may intensify the systemic inflammatory response by activating pro-inflammatory signaling pathways (like NF-kB), and it may also change the metabolic preferences of immune cells (like the polarization of macrophages to the pro-inflammatory M1 phenotype), thereby weakening the body’s anti-infection ability and delaying the repair of tissues and organs (59). Finally, due to the excessive supplementation of proteins, causing amino acid overload in the body, it may interfere with the autophagy process of the human body, affect the ability of cells to clear damaged proteins and pathogens, and thus be unfavorable for the control of infection and the recovery of organ functions (59). Some studies have also pointed out the “time window” hypothesis of nutritional support, that is, in the early stage (like the acute stage), high-protein intake may be required due to high catabolism to maintain nitrogen balance, but after entering the recovery stage, excessive protein supplementation may no longer be beneficial or even harmful (60). The findings of these research results have significant guiding significance for clinical practice, especially in the formulation of individualized nutritional support strategies.

Additionally, we also conducted an umbrella review and found that there was no statistically significant difference detected in mortality, mechanical ventilation time, length of hospital stay, and length of ICU stay. However, it should be noted that from a methodological perspective, umbrella reviews tend to focus on presenting the overall evidence landscape (“evidence map”) rather than directly validating specific interventions. Therefore, their results should mainly serve as supplementary integration of existing evidence. On this basis, we included meta-analyses based on randomized controlled trials to integrate the existing evidence. Compared with the strict protein intake threshold set in this study (≥1.2 g/kg/day vs. < 1.2 g/kg/day, with a difference of ≥0.2 g/kg/day), the inclusion criteria were relatively broad. This strategy referred to the common practices of previous umbrella reviews to expand the evidence coverage (61, 62). At the same time, the included meta-analyses had a certain degree of overlap, and the CCA was relatively high. This overlap mainly reflected the repeated inclusion of underlying trials rather than the addition of independent evidence, which may reduce the independence of the evidence and amplify the effect of the results. Therefore, the results in this part should be interpreted with caution. Nevertheless, to ensure the completeness of the review, we still retained it, but the related conclusions need to be carefully understood in combination with the above limitations. Meanwhile, due to the existing problems like the small number and uneven quality of current studies in this aspect, we did not conduct the corresponding subgroup analysis. More high-quality research is still needed in the future to improve our research.

### Strength and limitations

The advantage of our conducting this research lies in the following. Firstly, we carried out extensive and comprehensive retrieval and analysis of the articles and obtained the necessary information through various channels like Origin64. Secondly, in terms of data processing, we adopted a special approach. For instance, Python 3.10 ensured the consistency of all data forms in the article as much as possible, facilitating observation and comparison. Furthermore, the use of TSA can better help us detect the risk of type 1 or type 2 errors in the research results (31), and determine whether more research is needed to draw definite conclusions. The application of GRADE ensures the reliability of the research conclusion for us. Finally, based on the original meta-analyses, we also integrated the umbrella review, which not only filled the current research gap in this aspect, but also conducted a secondary comprehensive analysis of multiple meta-analyses related to this topic, significantly improving the evidence level of the article and making the conclusion more persuasive. However, at the same time, this article also has certain limitations. Firstly, a major limitation is that most of the included studies are of medium quality and small scale, which leads to a relatively small total number of participants in this study and may pose a risk of bias. Secondly, the inclusion criterion for protein intake was set at ≥1.2 g/kg/day. To harmonize the diverse reporting methods across randomized controlled trials, studies reporting intake in g/day were converted using a standard weight of 60 kg. We acknowledge that this is a methodological simplification adopted for feasibility and the limitation of not accounting for regional variations in body weight. Additionally, the TSA results indicate that the required information size was not reached for some outcomes in the present meta-analysis, thereby underscoring the need for more trials to draw firm conclusions. Moreover, the high CCA is a limitation, as it suggests a lack of independent evidence and could lead to an overestimation of the true effect. Furthermore, due to the small number of meta-analyses in our research direction, the number of high-quality articles included in the umbrella review was relatively small. This limitation was further complicated by the methodological diversity of the included meta-analyses (those by Van Ruijven and Castro, which incorporated study designs beyond RCTs) (10, 11), potentially introducing heterogeneity and limiting the robustness of our conclusions. Finally, since the evidence certainty of most results was evaluated as moderate, it is highly necessary to have more high-quality research. As summarized, considering these limitations, more robust studies are needed in the future to examine the relationship between appropriate protein supplementation doses and key clinical outcomes in critically ill patients.

Our future research should also focus on the different responses of critically ill patients with different disease(e.g., sepsis, trauma, postoperative, etc.) to protein metabolism and the corresponding response degrees (59), and dynamically evaluate the protein tolerance of critically ill patients in combination with biomarkers (e.g., urea nitrogen, inflammatory factors, etc.) (52). Although the results of this meta-analysis suggest that higher protein supplementation in critically ill patients has no significant impact on their related clinical outcomes, the results provide an important basis for the design of future clinical trials. This protocol is well-structured and well-designed. The implementation of this study will represent the latest and most comprehensive research in the field of protein supplementation for critically ill patients, especially the optimization of intervention strategies for high-risk populations.

## Conclusion

In conclusion, our systematic review, meta-analysis, and umbrella review studies suggest that higher protein intake did not show significant overall benefits, and there may be possible harmful signals in the longer-duration subgroups. The certainty of these signals remains uncertain and requires further confirmation in future high-quality trials. However, our findings suggested that long term high-protein intervention was associated with increased overall mortality, although this finding should be interpreted with caution and requires further confirmation, short and medium-term high-protein intervention was associated with reduced length of hospital stay in critically ill patients, although the robustness of this result remains uncertain, and length of ICU stay was shortened among low and medium-risk patients receiving the high-protein intervention, which should also be interpreted cautiously given the limited evidence. In addition, our use of umbrella review analyses, although not yielding significant positive results, may be related to the small number of included studies as well as their low quality, and partially addresses the current gap in this area. Future studies of higher quality are needed to further clarify the relationship between high-protein supplementation and clinical outcomes in critically ill patients.

## Data Availability

The original contributions presented in the study are included in the article/Supplementary material, further inquiries can be directed to the corresponding authors.

## Glossary

RCT: Randomized controlled trial
PRISMA: Preferred reporting items for systematic reviews and meta-analyses
ICU: Intensive care unit
PICOS: Population, intervention, comparison, outcomes, and study design
BMI: Body mass index
APACHE II: Acute Physiology and Chronic Health Evaluation II
SOFA: Sequential organ failure assessment
SAPS II: Simplified acute physiology score II
ROB 2.0: Risk of bias 2.0
AMSTAR-2: A measure tool to assess systematic reviews-2
EN: Enteral nutrition
PN: Parenteral nutrition
MD: Mean difference
CI: Confidence interval
OR: Odds ratio
RR: Relative risk
CCA: Corrected coverage areas
TSA: Trial sequential analysis
RIS: Required information size
GRADE: The Grading of recommendations assessment, development and evaluation
TBI: Traumatic brain injury
SD: Standard deviation
HR: Hazard ratio
CE: Cycle ergometry
