# Supplementary material for: High-protein supplementation in critically ill patients: a systematic review, meta-analysis and umbrella review of existing evidence
Source: Front Nutr. 2026 May 21;13:1788894. doi: 10.3389/fnut.2026.1788894 (PMC13233266; doi:10.3389/fnut.2026.1788894)
Supplement: Supplementary file 1 [file Table_1.DOCX]

**Additional file 1**

**List of Additional file 1**

[**additional file A(Tables)** 4](#_Toc204379427)

[**Table 1 Population, Intervention, Comparison, Outcome, Study design (PICOS) framework** 5](#_Toc204379428)

[**Table 2 Search strategy of PubMed for randomized control trials** 5](#_Toc204379429)

[**Table 3 Search strategy of Web of Science for randomized control trials** 6](#_Toc204379430)

[**Table 4 Search strategy of Embase for randomized control trials** 7](#_Toc204379431)

[**Table 5 Search strategy of Cochrane Library for randomized control trials** 8](#_Toc204379437)

[**Table 6 Search strategy of PubMed for umbrella review** 9](#_Toc204379438)

[**Table 7 Search strategy of Web of Science for umbrella review** 10](#_Toc204379439)

[**Table 8 Search strategy of Embase for umbrella review** 11](#_Toc204379440)

[**Table 9 Search strategy of Cochrane Library for umbrella review** 12](#_Toc204379446)

[**Table 10 Characteristics of included RCTs assessing effect of high-protein and conventional protein supplementation for critically ill patients** 13](#_Toc204379447)

[**Table 10 Characteristics of included RCTs assessing effect of high-protein and conventional protein supplementation for critically ill patients(continued)** 14](#_Toc204379448)

[**Table 11 Characteristics of included umbrella review assessing effect of high-protein and conventional protein supplementation for critically ill patients** 16](#_Toc204379449)

[**Table 12 Other information of included umbrella review assessing effect of high-protein and conventional protein supplementation for critically ill patients** 17](#_Toc204379450)

[**Table 13 The mortality outcome summary of the included RCTs** 18](#_Toc204379451)

[**Table 14 Other outcomes summary of RCTs** 19](#_Toc204379452)

[**Table 15 The mortality outcome summary of meta-analyses** 20](#_Toc204379453)

[**Table 16 Other outcomes summary of meta-analyses** 21](#_Toc204379454)

[**Table 17 The AMSTAR2 assessments** 23](#_Toc204379455)

[**Table 18 Overall mortality(subgroup analysis of short- and medium-term intervention VS long term intervention) by Stata 18.0** 24](#_Toc204379456)

[**Table 19 Corrected Covered Area(CCA)** 25](#_Toc204379457)

[**additional file B1(Figures)** 27](#_Toc204379458)

[**Fig. 1 Risk of bias summary of ROB 2.0** 28](#_Toc204379459)

[**Fig. 2 The subgroup analysis of overall mortality** 29](#_Toc204379460)

[**Fig. 3 Secondary outcomes** 30](#_Toc204379461)

[**additional file C(Others)** 31](#_Toc204379462)

[**additional file C1** 32](#_Toc204379463)

[**additional file C2** 33](#_Toc204379464)

**additional file A(Tables)**

**Table 1 Population, Intervention, Comparison, Outcome, Study design (PICOS) framework**

| **PICOS** | **Searched items** |
| --- | --- |
| Population | Critical Care; Intensive Care Units; Critical Illness |
| Interventions | Diet, High-Protein; High Protein |
| Comparisons | Low-Protein; conventional protein supplementation; conventional protein |
| Outcomes |  |
| Primary outcomes | The overall mortality rate; if the overall mortality rate is not reported, the 28-day, 90-day, or ICU or hospital mortality rate can be used as substitutes. |
| Secondary outcomes | Other mortality (e.g.,28-day mortality, 60-day mortality, Hospital mortality, and ICU mortality), length of ICU stay and length of hospital stay, adverse event rate (e.g.,diarrhea and pneumonia infection), mechanical ventilation time. |
| Studies design | Random control study, Control study, Random control trial, Clinical control trial, Clinical trial, Randomized control trial, Randomized controlled trial |

**Table 2 Search strategy of PubMed for randomized control trials**

| **Searched strategy in PubMed** | | |
| --- | --- | --- |
| Block 1: Citical Care | | |
| #1 | | (((((((("Critical Care"[Mesh]) OR (intensive therapy[Title/Abstract])) OR (therapy, intensive[Title/Abstract])) OR (intensive care[Title/Abstract])) OR (Care, Critical[Title/Abstract])) OR (Surgical Intensive Care[Title/Abstract])) OR (Care, Surgical Intensive[Title/Abstract])) OR (Intensive Care, Surgical[Title/Abstract])) OR (care, intensive[Title/Abstract]) |
| Block 2: Intensive Care Units | | |
| #2 | | (((((((((((((((((((((((("Intensive Care Units"[Mesh]) OR (close attention unit[Title/Abstract])) OR (critical care unit[Title/Abstract])) OR (general ICU[Title/Abstract])) OR (GICU[Title/Abstract])) OR (GICUs[Title/Abstract])) OR (ICU's[Title/Abstract])) OR (intensive care department[Title/Abstract])) OR (intensive therapy unit[Title/Abstract])) OR (intensive treatment unit[Title/Abstract])) OR (medical-surgery ICU[Title/Abstract])) OR (medical/surgical ICU[Title/Abstract])) OR (medical/surgical ICUs[Title/Abstract])) OR (medico-surgical ICU[Title/Abstract])) OR (respiratory care unit[Title/Abstract])) OR (respiratory care units[Title/Abstract])) OR (special care unit[Title/Abstract])) OR (surgery/medical ICU[Title/Abstract])) OR (surgical-medical ICUs[Title/Abstract])) OR (surgical/medical ICU[Title/Abstract])) OR (unit, intensive care[Title/Abstract])) OR (intensive care unit[Title/Abstract])) OR (ICU Intensive Care Units[Title/Abstract])) OR (ICU[Title/Abstract])) OR (ICUs[Title/Abstract]) |
| Block 3: Critical Illness | | |
| #3 | | (((("Critical Illness"[Mesh]) OR (Illness, Critical[Title/Abstract])) OR (Illnesses, Critical[Title/Abstract])) OR (Critically Ill[Title/Abstract])) OR (Critical Illnesses[Title/Abstract]) |
| Block 4: Participants | | |
| #4 | | #1 OR #2 OR #3 |
| Block 5: Intervention in experimental group | | |
| #5 | | ((((((((((("Diet, High-Protein"[Mesh]) OR (diet, protein[Title/Abstract])) OR (high protein diet[Title/Abstract])) OR (high-protein diet[Title/Abstract])) OR (protein meal[Title/Abstract])) OR (protein-enriched diet[Title/Abstract])) OR (protein-rich diet[Title/Abstract])) OR (protein diet[Title/Abstract])) OR (Diets, High-Protein[Title/Abstract])) OR (High-Protein Diets[Title/Abstract])) OR (high protein[Title/Abstract])) OR (diet, high protein[Title/Abstract]) |
| #6 | | High protein[Title/Abstract] |
| #7 | | #5 OR #6 |
| Block 6: Final merge | | |
| #8 | #4 AND #7 | |

Note: For a more comprehensive search, only P (Populations) and I (Interventions) were included in the search formula, and others in PICOS were excluded by literature screening.

**Table 3 Search strategy of Web of Science for randomized control trials**

| **Searched strategy in Web of Science** | | |
| --- | --- | --- |
| Block 1: Citical Care | | |
| #1 | | ((((((((TS=(care, intensive)) OR TS=(critical care)) OR TS=(intensive therapy)) OR TS=(therapy, intensive)) OR TS=(intensive care)) OR TS=(Care, Critical)) OR TS=(Surgical Intensive Care)) OR TS=(Care, Surgical Intensive)) OR TS=(Intensive Care, Surgical) |
| Block 2: Intensive Care Units | | |
| #2 | | ((((((((((((((((((((((((TS=(close attention unit)) OR TS=(critical care unit)) OR TS=(general ICU)) OR TS=(GICU)) OR TS=(GICUs)) OR TS=(ICU's)) OR TS=(intensive care department)) OR TS=(intensive care units)) OR TS=(intensive therapy unit)) OR TS=(intensive treatment unit)) OR TS=(medical-surgery ICU)) OR TS=(medical/surgical ICU)) OR TS=(medical/surgical ICUs)) OR TS=(medico-surgical ICU)) OR TS=(respiratory care unit)) OR TS=(respiratory care units)) OR TS=(special care unit)) OR TS=(surgery/medical ICU)) OR TS=(surgical-medical ICUs)) OR TS=(surgical/medical ICU)) OR TS=(unit, intensive care)) OR TS=(unit, intensive care)) OR TS=(ICU Intensive Care Units)) OR TS=(ICU)) OR TS=(ICUs) |
| Block 3: Critical Illness | | |
| #3 | | ((((TS=(Illness, Critical)) OR TS=(Illnesses, Critical)) OR TS=(Critically Ill)) OR TS=(Critical Illnesses)) OR TS=(Critical Illness) |
| Block 4: Participants | | |
| #4 | | #1 OR #2 OR #3 |
| Block 5: Intervention in experimental group | | |
| #5 | | (((((((((((TS=(diet, high protein)) OR TS=(diet, high-protein)) OR TS=(diet, protein)) OR TS=(high protein diet)) OR TS=(high-protein diet)) OR TS=(protein meal)) OR TS=(protein-enriched diet)) OR TS=(protein-rich diet)) OR TS=(protein diet)) OR TS=(Diets, High-Protein)) OR TS=(High-Protein Diets)) OR TS=(high protein) |
| Block 6: Final merge | | |
| #6 | #4 AND #5 | |

Note: For a more comprehensive search, only P (Populations) and I (Interventions) were included in the search formula, and others in PICOS were excluded by literature screening.

**Table 4 Search strategy of Embase for randomized control trials**

| **Searched strategy in Embase** | | |
| --- | --- | --- |
| Block 1: Citical Care | | |
| #1 | | 'intensive care'/exp OR 'care, intensive':ab,ti OR 'critical care':ab,ti OR 'intensive therapy':ab,ti OR 'therapy, intensive':ab,ti OR 'care, critical':ab,ti OR 'surgical intensive care':ab,ti OR 'care, surgical intensive':ab,ti OR 'intensive care, surgical':ab,ti OR 'intensive care':ab,ti |
| Block 2: Intensive Care Units | | |
| #2 | | 'intensive care unit'/exp OR 'close attention unit':ab,ti OR 'critical care unit':ab,ti OR 'general icu':ab,ti OR gicu:ab,ti OR gicus:ab,ti OR 'intensive care department':ab,ti OR 'intensive care units':ab,ti OR 'intensive therapy unit':ab,ti OR 'intensive treatment unit':ab,ti OR 'medical-surgery icu':ab,ti OR 'medical/surgical icu':ab,ti OR 'medical/surgical icus':ab,ti OR 'medico-surgical icu':ab,ti OR 'respiratory care unit':ab,ti OR 'respiratory care units':ab,ti OR 'special care unit':ab,ti OR 'surgery/medical icu':ab,ti OR 'surgical-medical icus':ab,ti OR 'surgical/medical icu':ab,ti OR 'unit, intensive care':ab,ti OR 'icu intensive care units':ab,ti OR icu:ab,ti OR icus:ab,ti OR 'intensive care unit':ab,ti |
| Block 3: Critical Illness | | |
| #3 | | 'critical illness'/exp OR 'illness, critical':ab,ti OR 'illnesses, critical':ab,ti OR 'critically ill':ab,ti OR 'critical illnesses':ab,ti OR 'critical illness':ab,ti |
| Block 4: Participants | | |
| #4 | | #1 OR #2 OR #3 |
| Block 5: Intervention in experimental group | | |
| #5 | | 'protein diet'/exp OR 'diet, high protein':ab,ti OR 'diet, high-protein':ab,ti OR 'diet, protein':ab,ti OR 'high protein diet':ab,ti OR 'high-protein diet':ab,ti OR 'protein meal':ab,ti OR 'protein-enriched diet':ab,ti OR 'protein-rich diet':ab,ti OR 'diets, high-protein':ab,ti OR 'high-protein diets':ab,ti OR 'high protein':ab,ti OR 'protein diet':ab,ti |
| #6 | | 'high protein':ab,ti |
| #7 | | #5 OR #6 |
| Block 6: Final merge | | |
| #8 | #4 AND #7 | |

Note: For a more comprehensive search, only P (Populations) and I (Interventions) were included in the search formula, and others in PICOS were excluded by literature screening.

**Table 5 Search strategy of Cochrane Library for randomized control trials**

| **Searched strategy in Cochrane Library** | | |
| --- | --- | --- |
| Block 1: Citical Care | | |
| #1 | | MeSH descriptor: [Critical Care] explode all trees |
| #2 | | (Critical Care):ti,ab,kw OR (intensive therapy):ti,ab,kw OR (therapy, intensive):ti,ab,kw OR (intensive care):ti,ab,kw OR (Care, Critical):ti,ab,kw |
| #3 | | (Surgical Intensive Care):ti,ab,kw OR (Care, Surgical Intensive):ti,ab,kw OR (Intensive Care, Surgical):ti,ab,kw OR (care, intensive):ti,ab,kw |
| #4 | | #1 OR #2 OR #3 |
| Block 2: Intensive Care Units | | |
| #5 | | MeSH descriptor: [Intensive Care Units] explode all trees |
| #6 | | (Intensive Care Units):ti,ab,kw OR (close attention unit):ti,ab,kw OR (critical care unit):ti,ab,kw OR (general ICU):ti,ab,kw OR (GICU):ti,ab,kw |
| #7 | | (GICUs):ti,ab,kw OR (ICU's):ti,ab,kw OR (intensive care department):ti,ab,kw OR (intensive therapy unit):ti,ab,kw OR (intensive treatment unit):ti,ab,kw |
| #8 | | (ICUs):ti,ab,kw OR (ICU):ti,ab,kw OR (ICU Intensive Care Units):ti,ab,kw OR (intensive care unit):ti,ab,kw OR (unit, intensive care):ti,ab,kw |
| #9 | | (special care unit):ti,ab,kw OR (respiratory care units):ti,ab,kw OR (respiratory care unit):ti,ab,kw |
| #10 | | #5 OR #6 OR #7 OR #8 OR #9 |
| Block 3: Critical Illness | | |
| #11 | | MeSH descriptor: [Critical Illness] explode all trees |
| #12 | | (Critical Illness):ti,ab,kw OR (Illness, Critical):ti,ab,kw OR (Illnesses, Critical):ti,ab,kw OR (Critically Ill):ti,ab,kw OR (Critical Illnesses):ti,ab,kw |
| #13 | | #11 OR #12 |
| Block 4: Participants | | |
| #14 | | #4 OR #10 OR #13 |
| Block 5: Intervention in experimental group | | |
| #15 | | MeSH descriptor: [Diet, High-Protein] explode all trees |
| #16 | | (Diet, High-Protein):ti,ab,kw OR (diet, protein):ti,ab,kw OR (high protein diet):ti,ab,kw OR (high-protein diet):ti,ab,kw OR (protein meal):ti,ab,kw |
| #17 | | (protein-enriched diet):ti,ab,kw OR (protein-rich diet):ti,ab,kw OR (protein diet):ti,ab,kw OR (Diets, High-Protein):ti,ab,kw OR (High-Protein Diets):ti,ab,kw |
| #18 | | (high protein):ti,ab,kw OR (diet, high protein):ti,ab,kw |
| #19 | | #15 OR #16 OR #17 OR #18 |
| Block 6: Final merge | | |
| #20 | #14 AND #19 | |

Note: For a more comprehensive search, only P (Populations) and I (Interventions) were included in the search formula, and others in PICOS were excluded by literature screening.

**Table 6 Search strategy of PubMed for umbrella review**

| **Searched strategy in PubMed** | | |
| --- | --- | --- |
| Block 1: Citical Care | | |
| #1 | | (((((((("Critical Care"[Mesh]) OR (intensive therapy[Title/Abstract])) OR (therapy, intensive[Title/Abstract])) OR (intensive care[Title/Abstract])) OR (Care, Critical[Title/Abstract])) OR (Surgical Intensive Care[Title/Abstract])) OR (Care, Surgical Intensive[Title/Abstract])) OR (Intensive Care, Surgical[Title/Abstract])) OR (care, intensive[Title/Abstract]) |
| Block 2: Intensive Care Units | | |
| #2 | | (((((((((((((((((((((((("Intensive Care Units"[Mesh]) OR (close attention unit[Title/Abstract])) OR (critical care unit[Title/Abstract])) OR (general ICU[Title/Abstract])) OR (GICU[Title/Abstract])) OR (GICUs[Title/Abstract])) OR (ICU's[Title/Abstract])) OR (intensive care department[Title/Abstract])) OR (intensive therapy unit[Title/Abstract])) OR (intensive treatment unit[Title/Abstract])) OR (medical-surgery ICU[Title/Abstract])) OR (medical/surgical ICU[Title/Abstract])) OR (medical/surgical ICUs[Title/Abstract])) OR (medico-surgical ICU[Title/Abstract])) OR (respiratory care unit[Title/Abstract])) OR (respiratory care units[Title/Abstract])) OR (special care unit[Title/Abstract])) OR (surgery/medical ICU[Title/Abstract])) OR (surgical-medical ICUs[Title/Abstract])) OR (surgical/medical ICU[Title/Abstract])) OR (unit, intensive care[Title/Abstract])) OR (intensive care unit[Title/Abstract])) OR (ICU Intensive Care Units[Title/Abstract])) OR (ICU[Title/Abstract])) OR (ICUs[Title/Abstract]) |
| Block 3: Critical Illness | | |
| #3 | | (((("Critical Illness"[Mesh]) OR (Illness, Critical[Title/Abstract])) OR (Illnesses, Critical[Title/Abstract])) OR (Critically Ill[Title/Abstract])) OR (Critical Illnesses[Title/Abstract]) |
| Block 4: Participants | | |
| #4 | | #1 OR #2 OR #3 |
| Block 5: Intervention in experimental group | | |
| #5 | | ((((((((((("Diet, High-Protein"[Mesh]) OR (diet, protein[Title/Abstract])) OR (high protein diet[Title/Abstract])) OR (high-protein diet[Title/Abstract])) OR (protein meal[Title/Abstract])) OR (protein-enriched diet[Title/Abstract])) OR (protein-rich diet[Title/Abstract])) OR (protein diet[Title/Abstract])) OR (Diets, High-Protein[Title/Abstract])) OR (High-Protein Diets[Title/Abstract])) OR (high protein[Title/Abstract])) OR (diet, high protein[Title/Abstract]) |
| #6 | | High protein[Title/Abstract] |
| #7 | | #5 OR #6 |
| Block 6:study type | | |
| #8 | (meta-analysis[Filter] OR systematicreview[Filter]) | |
| Block 7: Final merge | | |
| #9 | #4 AND #7 AND #8 | |

Note: For a more comprehensive search, only P (Populations) , I (Interventions) and S (Studies design) were included in the search formula, and others in PICOS were excluded by literature screening.

**Table 7 Search strategy of Web of Science for umbrella review**

| **Searched strategy in Web of Science** | | |
| --- | --- | --- |
| Block 1: Citical Care | | |
| #1 | | ((((((((TS=(care, intensive)) OR TS=(critical care)) OR TS=(intensive therapy)) OR TS=(therapy, intensive)) OR TS=(intensive care)) OR TS=(Care, Critical)) OR TS=(Surgical Intensive Care)) OR TS=(Care, Surgical Intensive)) OR TS=(Intensive Care, Surgical) |
| Block 2: Intensive Care Units | | |
| #2 | | ((((((((((((((((((((((((TS=(close attention unit)) OR TS=(critical care unit)) OR TS=(general ICU)) OR TS=(GICU)) OR TS=(GICUs)) OR TS=(ICU's)) OR TS=(intensive care department)) OR TS=(intensive care units)) OR TS=(intensive therapy unit)) OR TS=(intensive treatment unit)) OR TS=(medical-surgery ICU)) OR TS=(medical/surgical ICU)) OR TS=(medical/surgical ICUs)) OR TS=(medico-surgical ICU)) OR TS=(respiratory care unit)) OR TS=(respiratory care units)) OR TS=(special care unit)) OR TS=(surgery/medical ICU)) OR TS=(surgical-medical ICUs)) OR TS=(surgical/medical ICU)) OR TS=(unit, intensive care)) OR TS=(unit, intensive care)) OR TS=(ICU Intensive Care Units)) OR TS=(ICU)) OR TS=(ICUs) |
| Block 3: Critical Illness | | |
| #3 | | ((((TS=(Illness, Critical)) OR TS=(Illnesses, Critical)) OR TS=(Critically Ill)) OR TS=(Critical Illnesses)) OR TS=(Critical Illness) |
| Block 4: Participants | | |
| #4 | | #1 OR #2 OR #3 |
| Block 5: Intervention in experimental group | | |
| #5 | | (((((((((((TS=(diet, high protein)) OR TS=(diet, high-protein)) OR TS=(diet, protein)) OR TS=(high protein diet)) OR TS=(high-protein diet)) OR TS=(protein meal)) OR TS=(protein-enriched diet)) OR TS=(protein-rich diet)) OR TS=(protein diet)) OR TS=(Diets, High-Protein)) OR TS=(High-Protein Diets)) OR TS=(high protein) |
| Block 6: Study type | | |
| #6 | | TI=(meta) |
| Block 7: Final merge | | |
| #7 | #4 AND #5 AND #6 | |

Note: For a more comprehensive search, only P (Populations) , I (Interventions) and S (Studies design) were included in the search formula, and others in PICOS were excluded by literature screening.

**Table 8 Search strategy of Embase for umbrella review**

| **Searched strategy in Embase** | | |
| --- | --- | --- |
| Block 1: Citical Care | | |
| #1 | | 'intensive care'/exp OR 'care, intensive':ab,ti OR 'critical care':ab,ti OR 'intensive therapy':ab,ti OR 'therapy, intensive':ab,ti OR 'care, critical':ab,ti OR 'surgical intensive care':ab,ti OR 'care, surgical intensive':ab,ti OR 'intensive care, surgical':ab,ti OR 'intensive care':ab,ti |
| Block 2: Intensive Care Units | | |
| #2 | | 'intensive care unit'/exp OR 'close attention unit':ab,ti OR 'critical care unit':ab,ti OR 'general icu':ab,ti OR gicu:ab,ti OR gicus:ab,ti OR 'intensive care department':ab,ti OR 'intensive care units':ab,ti OR 'intensive therapy unit':ab,ti OR 'intensive treatment unit':ab,ti OR 'medical-surgery icu':ab,ti OR 'medical/surgical icu':ab,ti OR 'medical/surgical icus':ab,ti OR 'medico-surgical icu':ab,ti OR 'respiratory care unit':ab,ti OR 'respiratory care units':ab,ti OR 'special care unit':ab,ti OR 'surgery/medical icu':ab,ti OR 'surgical-medical icus':ab,ti OR 'surgical/medical icu':ab,ti OR 'unit, intensive care':ab,ti OR 'icu intensive care units':ab,ti OR icu:ab,ti OR icus:ab,ti OR 'intensive care unit':ab,ti |
| Block 3: Critical Illness | | |
| #3 | | 'critical illness'/exp OR 'illness, critical':ab,ti OR 'illnesses, critical':ab,ti OR 'critically ill':ab,ti OR 'critical illnesses':ab,ti OR 'critical illness':ab,ti |
| Block 4: Participants | | |
| #4 | | #1 OR #2 OR #3 |
| Block 5: Intervention in experimental group | | |
| #5 | | 'protein diet'/exp OR 'diet, high protein':ab,ti OR 'diet, high-protein':ab,ti OR 'diet, protein':ab,ti OR 'high protein diet':ab,ti OR 'high-protein diet':ab,ti OR 'protein meal':ab,ti OR 'protein-enriched diet':ab,ti OR 'protein-rich diet':ab,ti OR 'diets, high-protein':ab,ti OR 'high-protein diets':ab,ti OR 'high protein':ab,ti OR 'protein diet':ab,ti |
| #6 | | 'high protein':ab,ti |
| #7 | | #5 OR #6 |
| Block 7: Study type | | |
| #8 | | 'meta analysis'/de |
| Block 8: Final merge | | |
| #9 | #4 AND #7 AND #8 | |

Note: For a more comprehensive search, only P (Populations) , I (Interventions) and S (Studies design) were included in the search formula, and others in PICOS were excluded by literature screening.

**Table 9 Search strategy of Cochrane Library for umbrella review**

| **Searched strategy in Cochrane Library** | | |
| --- | --- | --- |
| Block 1: Citical Care | | |
| #1 | | MeSH descriptor: [Critical Care] explode all trees |
| #2 | | (Critical Care):ti,ab,kw OR (intensive therapy):ti,ab,kw OR (therapy, intensive):ti,ab,kw OR (intensive care):ti,ab,kw OR (Care, Critical):ti,ab,kw |
| #3 | | (Surgical Intensive Care):ti,ab,kw OR (Care, Surgical Intensive):ti,ab,kw OR (Intensive Care, Surgical):ti,ab,kw OR (care, intensive):ti,ab,kw |
| #4 | | #1 OR #2 OR #3 |
| Block 2: Intensive Care Units | | |
| #5 | | MeSH descriptor: [Intensive Care Units] explode all trees |
| #6 | | (Intensive Care Units):ti,ab,kw OR (close attention unit):ti,ab,kw OR (critical care unit):ti,ab,kw OR (general ICU):ti,ab,kw OR (GICU):ti,ab,kw |
| #7 | | (GICUs):ti,ab,kw OR (ICU's):ti,ab,kw OR (intensive care department):ti,ab,kw OR (intensive therapy unit):ti,ab,kw OR (intensive treatment unit):ti,ab,kw |
| #8 | | (ICUs):ti,ab,kw OR (ICU):ti,ab,kw OR (ICU Intensive Care Units):ti,ab,kw OR (intensive care unit):ti,ab,kw OR (unit, intensive care):ti,ab,kw |
| #9 | | (special care unit):ti,ab,kw OR (respiratory care units):ti,ab,kw OR (respiratory care unit):ti,ab,kw |
| #10 | | #5 OR #6 OR #7 OR #8 OR #9 |
| Block 3: Critical Illness | | |
| #11 | | MeSH descriptor: [Critical Illness] explode all trees |
| #12 | | (Critical Illness):ti,ab,kw OR (Illness, Critical):ti,ab,kw OR (Illnesses, Critical):ti,ab,kw OR (Critically Ill):ti,ab,kw OR (Critical Illnesses):ti,ab,kw |
| #13 | | #11 OR #12 |
| Block 4: Participants | | |
| #14 | | #4 OR #10 OR #13 |
| Block 5: Intervention in experimental group | | |
| #15 | | MeSH descriptor: [Diet, High-Protein] explode all trees |
| #16 | | (Diet, High-Protein):ti,ab,kw OR (diet, protein):ti,ab,kw OR (high protein diet):ti,ab,kw OR (high-protein diet):ti,ab,kw OR (protein meal):ti,ab,kw |
| #17 | | (protein-enriched diet):ti,ab,kw OR (protein-rich diet):ti,ab,kw OR (protein diet):ti,ab,kw OR (Diets, High-Protein):ti,ab,kw OR (High-Protein Diets):ti,ab,kw |
| #18 | | (high protein):ti,ab,kw OR (diet, high protein):ti,ab,kw |
| #19 | | #15 OR #16 OR #17 OR #18 |
| Block 6: Study type | | |
| #20 | | ("Meta"):ti,ab,kw OR ("meta analyses"):ti,ab,kw OR ("meta analysis"):ti,ab,kw |
| Block 7: Final merge | | |
| #21 | #14 AND #19 AND #20 | |

Note: For a more comprehensive search, only P (Populations) , I (Interventions) and S (Studies design) were included in the search formula, and others in PICOS were excluded by literature screening.

**Table 10 Characteristics of included RCTs assessing effect of high-protein and conventional protein supplementation for critically ill patients**

| **NO.** | **Author(publication year)** | **Country** | **Study year range** | **Study center** | **Nutritional strategy** | **Sample size**  I C | |
| --- | --- | --- | --- | --- | --- | --- | --- |
| 1 | A. Bukhari(2020) | Indonesia | 2017-2018 | Single-center | EN | 33 | 22 |
| 2 | A. R. H. van Zanten(2018) | The Netherlands, France, Belgium | 2016-2017 | Multi-center | EN+PN | 22 | 22 |
| 3 | C. A. Braunschweig(2015) | America | 2009-2013 | Single-center | EN+PN | 40 | 38 |
| 4 | D. D. Yeh(2020) | America, Jamaica | 2015-2017 | Multi-center | EN | 19 | 17 |
| 5 | D. K. Heyland(2023) | Argentina, Australia, Brazil, Canada, China, Greece, India, Iran, Japan, Malaysia, Mexico, Panama, Puerto Rico, Saudi Arabia, the United Kingdom, America | 2018-2021 | Multi-center | EN+PN | 645 | 656 |
| 6 | E. Dresen(2021) | Germany | 2017-2020 | Single-center | EN+PN | 21 | 21 |
| 7 | E. Uyar(2023) | Turkey | 2021-2022 | Single-center | EN | 25 | 24 |
| 8 | I. Kagan(2022) | Israel | 2013-2016 | Single-center | EN | 14 | 14 |
| 9 | J. L. M. Bels(2024) | The Netherlands, Belgium | 2020-2023 | Multi-center | EN+PN | 470 | 465 |
| 10 | J. R. A. Azevedo(2019) | Brazil | 2016-2017 | Single-center | EN+PN | 57 | 63 |
| 11 | K. Fetterplace(2018) | Australia | 2015-2017 | Single-center | EN+PN | 30 | 30 |
| 12 | K. Nakamura(2021) | Japan | 2018-2019 | Single-center | EN+PN | 60 | 57 |
| 13 | L. A. S. Chapple(2021) | Australia,New Zealand | 2019 | Multi-center | EN+PN | 58 | 58 |
| 14 | L. Carteron(2021) | France | 2012-2019 | Single-center | EN | 100 | 95 |
| 15 | M. Danielis(2019) | Italy | 2017 | Single-center | EN+PN | 19 | 21 |
| 16 | M. J. Allingstrup(2017) | Denmark | 2013-2016 | Single-center | EN+PN | 100 | 99 |
| 17 | Q. Zhang(2022) | China | 2017-2018 | Single-center | EN | 20 | 21 |
| 18 | W. Xiong(2021) | China | 2019-2020 | Single-center | EN+PN | 27 | 26 |

Note: I: Intervention, C: Control; EN: Enteral Nutrition, PN: Parenteral Nutrition

**Table 10 Characteristics of included RCTs assessing effect of high-protein and conventional protein supplementation for critically ill patients(continued)**

| **Author(year)** | **Intervention time(d)/**  **Intervention duration**  **(S/L)** | **Age**  **I C** | | **Sex(male) (%)**  **I C** | | **SOFA score**  **I C** | | **APACHE II score**  **I C** | | **SAPS II**  **I C** | | **Critical illness severity**  **(1: Low-and medium-risk,**  **2:**  **High risk)** | **BMI(kg/m²)**  **I C** | | **Protein dosage(g/kg/d)**  **I C** | |
| --- | --- | --- | --- | --- | --- | --- | --- | --- | --- | --- | --- | --- | --- | --- | --- | --- |
| A. Bukhari(2020) | 3/S | Oversll: 44.30  (18.08)  Low:  TBI:41.6(20.11),Non-TBI:  42.44  (20.27);High:  TBI:  38.29  (18.35),Non-TBI:  50.25  (15.92) | Overall: 49.50  (17.69)  TBI:  29.4  (5.73),  Non-TBI  :55.41  (15.47) | NA | NA | Overall: 3.95  (2.12)  Low:  TBI:  4.2  (1.10);  Non-TBI  :3.44  (2.46);  High:  TBI:  4.43  (0.79),  Non-TBI  :4.0  (2.66) | Overall:  3.50  (2.09)  TBI:  4.6  (2.19),  Non-TBI  :3.18  (2.01) | Overall: 18.82(4.23)  Low:  TBI:  23.2(5.40),  Non-TBI:  17.33(3.24)  High:  TBI:  21.0(4.32),  Non-TBI:  17.33(3.31) | Overall: 16.82(6.75)  TBI:  19.2(8.44),Non-TBI:  16.12(6.30) | NA | NA | 1 | NA | NA | Overall: 101.5  (38.89)  Low:  TBI:  93.79  (26.65),  Non-TBI:  80.29  (29.38);  High:  TBI:  134.03  (42.58),Non-TBI:  102.88  (37.47) | Overall: 55.70  (36.89)  TBI:  57.12  (44.65),  Non-TBI:  55.28  (35.87) |
| A. R. H. van Zanten(2018) | 28/L | 63.9  (13.3) | 60.8  (15.2) | 40.9% | 59.1% | 10(9–11) | 9(7–11) | 25(21–28) | 24(18-27) | NA | NA | 1 | 30.3(4.1) | 30.7(8.4) | 1.54 | 0.80 |
| C. A. Braunschweig(2015) | ≈30/L | 52.5  (17.1) | 58.6  (16.2) | 51.2%  (female) | 44.7%  (female) | 9.3(3.8) | 9.4(3.4) | 23.4(9.3) | 27.7(7.9) | NA | NA | 2 | 29.8(9.3) | 30.1(8.9) | 82(23)  (k/d) | 60.4(24)  (k/d) |
| D. D. Yeh(2020) | 12/S | 49.1  (24.7) | 50.6  (15.6) | 95% | 71% | 7.3(3.9) | 8.8(3.4) | 19.6(7.3) | 17.5(10.1) | NA | NA | 1 | 28.5(6.7) | 30.2(6.1) | 1.2(0.4) | 0.9(0.4) |
| D. K. Heyland(2023) | 28/L | 57  (18–95) | 57  (18–95) | 61% | 59% | 9(6–11) | 9(6–11) | 21(16–27);  n=609 | 21(15–26);  n=621 | NA | NA | 1 | 28(13–85) | 29(13–77) | 1.6(0.5) | 0.9(0.3) |
| E. Dresen(2021) | 28/L | 66(16) | 64(15) | 71.4% | 71.4% | 6(3) | 8(3) | NA | NA | 46(12) | 45(10) | 2 | NA | NA | 1.5(0.5) | 1.0(0.4) |
| E. Uyar(2023) | 15/L | 61.72  (21.95) | 59.79  (23.82) | 76% | 50% | 8.08  (3.15) | 7.54  (2.28) | 30.52(11.27) | 33.21(8.64) | NA | NA | 2 | 22.18  (3.87) | 22.84  (4.41) | 2.2 | 1.2 (target protein) |
| I. Kagan(2022) | 14/S | 58(15) | 57(19) | 71.4% | 57.1% | 7(4) | 5(2) | 21(8) | 21(6) | NA | NA | 1 | 30(7) | 29(6) | 83.5  (24.7) | 70.9(22.7) |
| J. L. M. Bels(2024) | 90/L | 62(14) | 63(14) | 62% | 67% | 9(3) | 10(3) | 21(7) | 22(7) | NA | NA | 1 | 28(6) | 27(5) | 1.87  (0.96-2.00) | 1.19  (0.63–1.26) |
| J. R. A. Azevedo(2019) | 14/S | 65.0  (18.8) | 67.4  (18.9) | 40.3%  (female) | 49.2%  (female) | 9.8(14.6) | 6.8(4.0) | NA | NA | NA | NA | 2 | NA | NA | 1.69  (1.33-1.80) | 1.13  (0.97-1.34) |
| K. Fetterplace(2018) | 15/L | 55(13) | 57(16) | 77% | 70% | NA | NA | 22(6.2) | 20(5.9) | NA | NA | 1 | 30(7.1) | 29(5.3) | 1.20  (0.30) | 0.75(0.11) |
| K. Nakamura(2021) | 10/S | 68.3  (14.3) | 67.9  (14.9) | 58.3% | 66.7% | 6.8(3.1) | 7.1(3.5) | 18.6(8.1) | 18.2(6.0) | NA | NA | 1 | 21.3(3.9) | 21.5(4.5) | 1.5 | 0.8 |
| L. A. S. Chapple(2021) | 28/L | 60  (50-72) | 61  (46-68) | 67% | 76% | NA | NA | 22(16-26) | 22(16-27) | NA | NA | 1 | 29  (26-33) | 30  (25-34) | 1.52(0.52) | 0.99(0.27) |
| L. Carteron(2021) | 10/S | 57  (44–65) | 55  (40–65) | 67% | 56% | NA | NA | NA | NA | 48(12) | 49(13) | 2 | 26  (23–29) | 26  (23–29) | 1.3(0.4) | 1.1(0.3) |
| M. Danielis(2019) | <14/S | 66  (57-72) | 63  (46-70) | 58% | 52% | NA | NA | 17.0  (13.5-22.0) | 17.0  (13.0-22.0) | 32.0  (28.0-48.5) | 40.0  (31.0-48.0) | 1 | 25.0  (23.0-27.5) | 24.0  (21.0-26.0) | 117.84  (16.75)  (k/d) | 65.86  (19.70)  (k/d) |
| M. J. Allingstrup(2017) | 7/S | 63  (51–72) | 68  (52–75) | 65% | 60% | 8(6–11) | 8(5–10) | NA | NA | NA | NA | 2 | 22(20-26) | 22(20-25) | 1.47  (1.13-1.69) | 0.50  (0.29-0.69) |
| Q. Zhang(2022) | 35/L | 64.45  (16.17) | 69.24  (18.15) | 60% | 85.7% | 8.15  (3.08) | 8.14  (2.41) | 21.75(7.15) | 20.48(6.97) | NA | NA | 1 | 22.18  (3.87) | 22.84  (4.41) | 1.70  (0.21) | 1.06(0.21) |
| W. Xiong(2021) | 7/S | 49.44  (14.94) | 48.31  (10.41) | 74.07% | 65.38% | NA | NA | 21.41(2.14) | 21.27(2.22) | NA | NA | 1 | 21.58  (1.33) | 21.95  (1.40) | 1.2-1.7 | 0.5-0.7 |

Note: S/L: short- and medium-term intervention/long term intervention; Overall: the overall value after combining the mean and the standard deviation; Low: oligomeric group; High: high-protein polymeric group; TBI: traumatic brain injury; SOFA: Sequential Organ Failure Assessment; APACHE II: Acute Physiology and Chronic Health Evaluation II; SAPS II: Simplified Acute Physiology Score II; BMI: Body Mass Index; NA: Not Available

**Table 11 Characteristics of included umbrella review assessing effect of high-protein and conventional protein supplementation for critically ill patients**

| **Author(year)** | **Country** | **End search** | **Original research type** | **N** | **n** | **Patients** **definition** | **sex(male)**  **(%)** | **Range of mean ages(y)** | **Protein dosage (g/kg/d)**  **I C** | | **Outcome** | **Conflict of interest** |
| --- | --- | --- | --- | --- | --- | --- | --- | --- | --- | --- | --- | --- |
| I. M. van Ruijven  (2023) | Netherlands | 2022.10.19 | RCT、prospective study、prospective retrospective study、retrospective study | 29 | 7190 | Patients are 18 years with an ICU stay of 2 days | 37.1-95 | 48.3-72.5 | ≥1.2 | <1.2（a difference of 0.2 between two groups） | Hospital mortality, ICU mortality, 28-day mortality, mechanical ventilation time, length of ICU stay, length of hospital stay, infectious complications | None |
| M. L. Davies  (2017) | Australia | 2015.12.31 | RCT | 14 | 3238 | Comparing different nutritional support strategies (≥48 hours) in critically ill adult trials (≥50% mechanically ventilated | 37.3-76.7 | 48.0-70.9 | 1.02(0.42) | 0.67(0.38) | Overall mortality, mechanical ventilation time, length of ICU stay, length of hospital stay | NA |
| S. Castro  (2025) | Portugal | 2023.6.1 | RCT、retrospective study | 4 | 1730 | Adult critically patients admitted to ICU 7 days | NA | NA | >1.3 | ≤1.3 | Overall mortality | The author, Sílvia Castro, reports receiving speaker fees |
| S. Heuts  (2025) | Malaysia | 2024.9.4 | RCT | 22 | 4164 | critically ill adult patients treated in the ICU (critical illness was defined as mechanically ventilated or mortality of > 5% in the control group) | 38.46-100 | 32.7-74.2 | 1.5(0.6) | 0.9(0.4) | Overall mortality, mechanical ventilation time, length of hospital stay, infectious complications | NA |
| Y. G. Qin  (2024) | China | 2023.11.20 | RCT | 17 | 2965 | critically ill adult patients | NA | NA | 1.2-2.0 | 0.5-1.1 | Overall mortality, mechanical ventilation time, length of ICU stay, length of hospital stay | None |
| Z. Y. Lee  (2021) | Malaysia | 2021.4.1 | RCT | 19 | 1731 | Adult (age ≥ 18 years old) critically ill patients (mechanically ventilated or mortality of > 5% in the control group) | 38.46-100 | 34-74 | 1.31(0.48) | 0.90(0.30) | Overall mortality, hospital mortality, ICU mortality, 28-day mortality, mechanical ventilation time, length of ICU stay, length of hospital stay, infectious complications | None |
| Z. Y. Lee  (2024) | Malaysia | 2023.5.29 | RCT | 23 | 3303 | adult (age ≥ 18) critically ill patients (mechanically ventilated or if uncertain, the control group mortality had to be greater than 5% to ensure including truly critically ill patients) | 38.46-100 | 32.7-74 | 1.49(0.48) | 0.92(0.30) | Overall mortality, hospital mortality, ICU mortality, 28-day mortality, mechanical ventilation time, length of ICU stay, length of hospital stay, infectious complications | None |
| N. Nakanishi  (2022) | Japan | 2021.11.3 | RCT | 14 | 1690 | The included population was critically ill adult patients (≥18 years of age)  admitted to the ICU. No specific diagnostic criteria were used for population selection. | NA | 44-73.9 | ≥1.0 | <1.0 | Overall mortality, length of hospital stay | None |

Note: N: the number of studies included in, n: the number of patients included in, y: year; NA: Not Available

**Table 12 Other information of included umbrella review assessing effect of high-protein and conventional protein supplementation for critically ill patients**

| **Author(year)** | **BMI range(****kg/m²)** | **APACHE II score range** | **Disease type** | **Author's Conclusion** |
| --- | --- | --- | --- | --- |
| I. M. van Ruijven  (2023) | 18.5-43.0 | 13.9-25.4 | NA | High protein provision of more than 1.2 g/kg in critically ill patients seemed to improve nitrogen balance and changes in muscle mass on the short-term and likely 60-day mortality. Data on long-term effects on quality of life are urgently needed. |
| M. L. Davies  (2017) | 22.8-30.1 | 18-27.7 | ALI/ARDS, Mixed, Surgical, Medical, Neurotrauma | Delivery of varying amounts of nutritional protein was not associated with any effect on mortality |
| S. Castro  (2025) | NA | NA | Surgical, medical | 28 ICU Support.After the first week of critical illness, increasing protein intake to >1.3 g/kg/d may improve early mortality but not late mortality or other clinical outcomes. The small number of relevant studies and the heterogeneity of outcomes assessed, weaken these conclusions. Further studies are warranted to discern whether higher protein intake is beneficial in chronic critical illness. |
| S. Heuts  (2025) | 21.3-30.7 | 28.5(22.3-32.8) | NA | There is a considerable probability of an increased mortality risk with higher protein delivery in critically ill patients, although a clinically beneficial effect cannot be completely eliminated based on the current data. |
| Y. G. Qin  (2024) | NA | NA | NA | For critically ill patients, the protein supplementation dose had no significant effect on clinical outcomes, including overall mortality, length ofintensive care unit and hospital stay, duration ofmechanical ventilation, and incidence ofacute kidney injury |
| Z. Y. Lee  (2021) | NA | 28.5(22.3-32.8) | Severe head injury, Severe stroke, Brain injured, Surgical, Mixed | In critically ill patients, a higher daily protein delivery was not associated with any improvement in clinical or patient‑centered outcomes. Larger, and more definitive RCTs are needed to confirm the effect of muscle loss attenuation associated with higher protein delivery |
| Z. Y. Lee  (2024) | 21.3-30.7 | 28.5(22.3-32.8) | Severe head injury, Severe stroke, Brain injured, Surgical, Mixed, SAH | Higher, compared with lower protein delivery, does not appear to affect clinical outcomes in general critically ill patients but may increase mortality rates in patients with AKI. Further investigation of the combined early physical rehabilitation intervention in non‑AKI patients is warranted |
| N. Nakanishi  (2022) | NA | NA | Infection, Neurocritical ICU, Mixed | The current meta-analysis revealed that high protein delivery >1.0 g/kg/day during the first 4 to 10 days of ICU was associated with attenuated muscle loss and slightly improved ADL, while high energy delivery did not exert significant effects. |

Note: BMI: Body Mass Index; NA: Not Available

**Table 13 The mortality outcome summary of the included RCTs**

| **Author(year)** | **Overall mortality (%)**  **I C** | | **28-day mortality (%)**  **I C** | | **60-day mortality (%)**  **I C** | | **Hospital mortality (%)**  **I C** | | **ICU mortality (%)**  **I C** | |
| --- | --- | --- | --- | --- | --- | --- | --- | --- | --- | --- |
| A. Bukhari(2020) |  |  |  |  |  |  | Overall:10  low:TBI:1(25%);  Non-TBI:2(20%)  high:TBI:4(57.1%);  Non-TBI:3(25%) | Overall:5 TBI:2(40%);  Non-TBI:3(17.6%) |  |  |
| A. R. H. van Zanten(2018) |  |  | 2(9.1%) | 3(13.6%) |  |  |  |  |  |  |
| C. A. Braunschweig(2015) | 16(40.0%) | 6(15.8%) |  |  |  |  |  |  |  |  |
| D. D. Yeh(2020) | 1(5%) | 2(12%) |  |  |  |  |  |  |  |  |
| D. K. Heyland(2023) |  |  |  |  | 222(34.6%);  n=642 | 208(32.1%);  n=648 | 211(32.8%) ;  n=644 | 188(28.8%);  n=653 |  |  |
| E. Dresen(2021) |  |  |  |  |  |  |  |  | 8(38%) | 7(33%) |
| E. Uyar(2023) |  |  | 6(24.0%) | 6(25.0%) |  |  |  |  |  |  |
| I. Kagan(2022) |  |  |  |  |  |  | 5(35.7%) | 4(28.6%) | 3(21.4%) | 3(21.4%) |
| J. L. M. Bels(2024) | 197(42%) | 177(38%) |  |  |  |  |  |  |  |  |
| J. R. A. Azevedo(2019) |  |  |  |  |  |  | 26(45.6%) | 29(46.0%) | 22(38.5%) | 28(44.4%) |
| K. Fetterplace(2018) |  |  | 4(13%) | 5(17%) | 4(13%) | 5(17%) |  |  |  |  |
| K. Nakamura(2021) |  |  | 6(10%) | 6(10.5%) |  |  |  |  |  |  |
| L. A. S. Chapple(2021) |  |  | 12/56  (21%) | 14/57  (25%) |  |  |  |  | 12/58(21%) | 10/58(17%) |
| L. Carteron(2021) |  |  | 20(20%) | 21(22%) | 23(23%) | 23(24%) |  |  |  |  |
| M. Danielis(2019) | 2(11%) | 7(33%) |  |  |  |  |  |  |  |  |
| M. J. Allingstrup(2017) |  |  | 20(20%) | 21(21%) |  |  |  |  |  |  |
| Q. Zhang(2022) |  |  |  |  |  |  |  |  | 4(20%) | 6(28.57%) |
| W. Xiong(2021) |  |  | 8(29%) | 11(42%) |  |  |  |  |  |  |

Note: Overall: the overall value after combining the mean and the standard deviation; Low: oligomeric group; High: high-protein polymeric group; TBI: traumatic brain injury

**Table 14 Other outcomes summary of RCTs**

| **Author(year)** | **length of ICU stay**  **I C** | | **length of hospital stay**  **I C** | | **mechanical ventilation time(d)**  **I C** | | **diarrhea(%)**  **I C** | | **pneumonia infection(%)**  **I C** | | **Insulin requirement(IU)**  **I C** | | **RRT(%)**  **I C** | |
| --- | --- | --- | --- | --- | --- | --- | --- | --- | --- | --- | --- | --- | --- | --- |
| A. Bukhari(2020) | Overall:  9.93(7.80)  Low:TBI:  17.33(9.71),  Non-TBI:  6.00(3.51);  High:TBI:  15.33(9.02),  Non-TBI:  7.60(6.20) | Overall:  11.84(26.51) TBI:  35.33(52.58),  Non-TBI:  4.93(2.82) | Overall:  21.84(14.65) Low:TBI:  44.33(27.39),  Non-TBI:  17.38(6.95);  High:TBI:  24.33(10.21),  Non-TBI:  16.60(9.35) | Overall:  20.74(30.63)  TBI:46.33  (60.50),  Non-TBI:  13.21(7.17) |  |  |  |  |  |  |  |  |  |  |
| A. R. H. van Zanten(2018) | 18.4(13.4) | 18.3(12.7) | 28.5(13.3) | 28.2(13.2) | 10.0(8.7) | 7.4(5.4) | 8(36.4%) | 11(50.0%) |  |  |  |  |  |  |
| C. A. Braunschweig(2015) | 15.5(12.8) | 16.1(11.5) | 27.2(18.2) | 22.8(14.3) | 6(4-10) | 7(3-14) |  |  |  |  | 23.6  (47.6) | 14  (23.6) |  |  |
| D. D. Yeh(2020) | 12.0  (6.0–41.7) | 12.5  (10.6-15.9) | 21.4  (14.9-60.0) (N=18) | 16.6  (13.9-21.1) (N=15) |  |  |  |  |  |  |  |  |  |  |
| D. K. Heyland(2023) | 10.0  (5.6-18.2)  (N=642) | 9.4  (5.1-19.2) (N=650) | 19.3  (9.7-39.0) (N=642) | 18.9  (9.4-37.9) (N=647) | 6.1  (3.0-13.8) (N=645) | 6.1  (2.8-12.8) (N=656) |  |  |  |  | 44.9  (48.2) (N=  364) | 40.4  (41.1) (N=  365) |  |  |
| E. Dresen(2021) | 68(34) | 62(48) |  |  | 797(133)  (hour) | 758(191)  (hour) |  |  | 19  (90%) | 17  (81%) |  |  | 10  (48%) | 8  (38%) |
| E. Uyar(2023) |  |  |  |  |  |  |  |  |  |  |  |  |  |  |
| I. Kagan(2022) | 20.8(10.1) | 19.3(7.2) | 36.2(27.4) | 30.7(17.9) | 14.9(9.4) | 15.8(7.1) |  |  |  |  |  |  |  |  |
| J. L. M. Bels(2024) |  |  |  |  |  |  |  |  | 111  /469  (24%) | 115  /465  (25%) |  |  |  |  |
| J. R. A. Azevedo(2019) | 21(13-33) | 18(10-35) |  |  | 9(5-14) | 9(5-14) |  |  |  |  |  |  |  |  |
| K. Fetterplace(2018) | 10.6(8.3) | 9.1(5.5) | 27.4(19.0) | 18.8(10.9) | 8.7(7.5) | 7.0(5.0) | 16(53%) | 16(53%) |  |  |  |  |  |  |
| K. Nakamura(2021) | 7  (5-12) | 9  (6-13) | 26.50  (18.00-58.00) | 45.50  (18.25-75.75) | 5(2-6.5) | 5.5(3-9) | 14(23.3%) | 19(33.3%) | 21  (35.6%) | 29  (50.9%) |  |  |  |  |
| L. A. S. Chapple(2021) | 13(13) | 14(18) | 24(21) | 26(32) |  |  |  |  |  |  |  |  | 12  (21%) | 13  (22%) |
| L. Carteron(2021) | 14(8-21) | 15(10-23) |  |  | 10(6-16) | 11(6-17) | 16(16%) | 8(8%) | 47  (47%) | 41  (43%) |  |  |  |  |
| M. Danielis(2019) |  |  |  |  | 5(4-12) | 8(6-13) |  |  |  |  |  |  |  |  |
| M. J. Allingstrup(2017) | 7(5-22) | 7(4-11) | 30(12-53) | 34(14-53) |  |  |  |  | 4  (4%) | 4  (4%) |  |  | 22  (22%) | 19  (17%) |
| Q. Zhang(2022) | 27.94(11.44) | 28.40(11.78) |  |  |  |  |  |  |  |  |  |  |  |  |
| W. Xiong(2021) | 10.95(10.00-12.48) | 12.97(11.17-14.96) | 14.95(12.97-16.96) | 17.96(14.21-21.90) | 22.59  (9.99) | 23.29  (11.81) |  |  |  |  |  |  |  |  |

Note: Overall: the overall value after combining the mean and the standard deviation; Low: oligomeric group; High: high-protein polymeric group; TBI: traumatic brain injury; RRT: renal replacement therapy

**Table 15 The mortality outcome summary of meta-analyses**

| **Author(year)** | **Overall mortality** | **28-day mortality** | **Hospital mortality** | **ICU mortality** |
| --- | --- | --- | --- | --- |
| I. M. van Ruijven  (2023) |  | RR, 0.90, 95% CI 0.65 to 1.25; I² = 54% | RR, 0.89, 95% CI 0.73 to 1.08; I² = 51% | RR, 0.89, 95 CI% 0.73 to 1.10, I² = 38% |
| M. L. Davies  (2017) | C/I  OR, 0.935; 95% CI, 0.716–1.219, I² = 48.2% |  |  |  |
| S. Castro  (2025) | HR(≈RR), 0.93; 95% CI, 0.76–1.15, I² = 80% |  |  |  |
| S. Heuts  (2025) | RR, 1.01; 95% CI, 0.84–1.16 |  |  |  |
| Y. G. Qin  (2024) | RR, 1.03, 95% CI, [0.92–1.15], I² = 0% |  |  |  |
| Z. Y. Lee  (2021) | RR, 0.91, 95% CI 0.75–1.10,I² = 0% | RR, 0.98, 95% CI 0.76–1.26, I² = 0% | RR, 0.94, 95% CI 0.74–1.20, I² = 0% | RR, 0.94, 95% CI 0.74–1.20, I² = 0% |
| Z. Y. Lee  (2024) | RR, 0.99, 95% CI 0.88–1.11, I² = 0% | RR, 0.83, 95% CI 0.60–1.15, I² = 0% | RR, 0.88, 95% CI 0.67–1.15, I² = 26% | RR, 0.85, 95% CI 0.69–1.05, I² = 0% |

Note: C/I: Indicate that this outcome is Favour Control/Favour Intervention. If not specified, other outcomes are defaulted to Favour Intervention/Favour Control

**Table 16 Other outcomes summary of meta-analyses**

| **Author(year)** | **Mechanical ventilation time(d)** | **Length of ICU stay** | **Length of hospital stay** | **Infectious complications** |
| --- | --- | --- | --- | --- |
| I. M. van Ruijven  (2023) | MD, 0.8, 95%CI -0.7 to 2.4, I² = 75% | MD, -0.0, 95% CI -1.7 to 1.7; I² = 80% | MD, 1.0, 95% CI -3.7 to 5.7, I² = 80% | RR, 0.74, 95% CI 0.74 to 1.86; I² = 70% |
| M. L. Davies  (2017) | C/I  MD, –0.073 days; 95% CI, –0.821 to 0.676 | C/I  MD, 0.039 days; 95% CI, –0.746 to 0.825 | C/I  MD, –0.963 days; 95% CI, –3.932 to 2.006 |  |
| S. Castro  (2025) |  |  |  |  |
| S. Heuts  (2025) | MD, –0.18, 95% CI, –0.87 to 0.59 |  |  | RR, 1.03; 95% CI, 0.84 to 1.23 |
| Y. G. Qin  (2024) | MD, −0.14, 95%CI [−0.83 to 0.54], I² = 8% | MD, 0.19, 95%CI [−0.67 to 1.04], I² = 25% | MD, 0.73, 95%CI [−1.59 to 3.04], I² = 27% |  |
| Z. Y. Lee  (2021) | MD, −0.57, 95% CI −1.29 to 0.14, I² = 8% | MD, −0.76, 95% CI −1.75 to 0.23, I² = 0% | MD, 0.34, 95% CI -3.32 to 4.00, I² = 22% | RR, 1.05, 95% CI 0.88–1.25, I² = 0% |
| Z. Y. Lee  (2024) | MD, −0.42, 95% CI −1.00 to 0.16, I² = 1% | MD, − 0.44, 95% CI − 1.27 to 0.39, I² = 0% | MD, 1.55, 95% CI − 0.55 to 3.65, I² = 18% | RR, 1.05, 95% CI 0.88–1.25 |

Note: C/I: Indicate that this outcome is Favour Control/Favour Intervention. If not specified, other outcomes are defaulted to Favour Intervention/Favour Control

**Table 17 The** **AMSTAR2 assessments**

| Author | M. L. Davies  (2017) | Z. Y. Lee  (2021) | I. M. van Ruijven  (2023) | Z. Y. Lee  (2024) | Y. G. Qin  (2024) | S.Castro  (2025) | S.Heuts  (2025) | Nobuto Nakanishi  (2022) |
| --- | --- | --- | --- | --- | --- | --- | --- | --- |
| 1.PICOY/N | Y | Y | Y | Y | Y | Y | Y | Y |
| 2.DeviationY/P/N | P | Y | Y | Y | Y | Y | Y | Y |
| 3.Selection of Study Y/N | Y | Y | Y | Y | Y | Y | Y | Y |
| 4.Comprehensive literature search Y/P/N | Y | Y | Y | Y | Y | P | Y | Y |
| 5.Duplicate selection Y/N | Y | Y | Y | Y | Y | Y | Y | Y |
| 6.Duplicated data extraction  Y/N | Y | Y | Y | Y | Y | Y | Y | Y |
| 7.List of excluded studies Y/P/N | Y | Y | P | Y | Y | Y | Y | Y |
| 8.Describe studies in detail Y/P/N | Y | Y | Y | Y | Y | Y | Y | Y |
| 9.Bias assessment  (RCT/NRSI) Y/P/N | Y | Y | Y | Y | Y | Y | Y | Y |
| 10.Source of individual funding Y/N | N | Y | Y | Y | N | N | N | Y |
| 11.Adequated statistical method  (Y/N/not MAs) | Y | Y | Y | Y | Y | Y | Y | Y |
| 12.RoB risk of bias in studies (Y/N/not MAs) | N | Y | Y | Y | N | N | Y | Y |
| 13.Discussion using risk of bias Y/N | Y | Y | Y | Y | Y | Y | Y | Y |
| 14.Explain heterogeneity  Y/N | Y | Y | Y | Y | Y | Y | Y | Y |
| 15.Publication bias (Y/N/not MAs) | Y | Y | N | Y | Y | N | Y | Y |
| 16.Conflict of interest Y/N | Y | Y | Y | Y | Y | Y | Y | Y |
| Interpret | Moderate | High | Low | High | Moderate | Critically low | High | Low |

Y: Yes, N: No, P: Partial, MA: meta-analysis

**Table 18 Overall mortality(subgroup analysis of short- and medium-term intervention VS long term intervention) by Stata 18.0**

Study | OR [95% Conf. Interval] % Weight

---------------------+---------------------------------------------------

Short-medium

A. Bukhari | 1.478 0.426 5.124 1.40

D. D. Yeh | 0.417 0.034 5.057 0.35

I. Kagan | 1.389 0.282 6.835 0.85

J. R. A. Azevedo | 0.786 0.379 1.629 4.07

K. Nakamura | 0.944 0.286 3.119 1.52

L. Carteron | 0.881 0.442 1.755 4.55

M. Danielis | 0.235 0.042 1.318 0.73

M. J. Allingstrup | 0.929 0.467 1.846 4.58

W. Xiong | 0.574 0.185 1.786 1.68

Sub-total |

D+L pooled OR | 0.840 0.603 1.169 19.72

---------------------+---------------------------------------------------

Long

A. R. H. van Zanten | 0.633 0.095 4.218 0.60

C. A. Braunschweig | 3.556 1.211 10.441 1.86

D. K. Heyland | 1.205 0.952 1.526 38.77

E. Dresen | 1.231 0.348 4.358 1.35

E. Uyar | 0.947 0.258 3.484 1.27

J. L. M. Bels | 1.174 0.904 1.526 31.52

K. Fetterplace | 0.769 0.185 3.198 1.07

L. A. S. Chapple | 0.838 0.348 2.016 2.80

Q. Zhang | 0.625 0.147 2.659 1.03

Sub-total |

D+L pooled OR | 1.181 1.002 1.391 80.28

---------------------+---------------------------------------------------

Overall |

D+L pooled OR | 1.104 0.953 1.279 100.00

---------------------+---------------------------------------------------

Test(s) of heterogeneity:

Heterogeneity degrees of

statistic freedom P I-squared** Tau-squared

1 4.18 8 0.841 0.0% 0.0000

2 6.26 8 0.618 0.0% 0.0000

Overall 13.70 17 0.688 0.0% 0.0000

** I-squared: the variation in OR attributable to heterogeneity)

Note: between group heterogeneity not calculated;

only valid with inverse variance method

Significance test(s) of OR=1

1 z= 1.03 p = 0.301

2 z= 1.98 p = 0.047

Overall z= 1.32 p = 0.187

**Table 19 Corrected Covered Area(CCA)**

| No | **Individual studies** | **M. L. Davies,2017** | **Z. Y. Lee,2021** | **I. M. van Ruijven,2023** | **Z. Y. Lee,2024** | **Y. G. Qin,2024** | **S. Castro,2025** | **S. Heuts,2025** | **Nobuto Nakanishi,2022** | **Overlap** |
| --- | --- | --- | --- | --- | --- | --- | --- | --- | --- | --- |
| 1 | **Nakamura K，2021** |  | 1 | 1 | 1 | 1 |  | 1 | 1 | 1 |
| 2 | **Dresen E，2021** |  | 1 | 1 | 1 | 1 | 1 | 1 |  | 1 |
| 3 | **Bukhari A，2020** |  | 1 |  | 1 |  |  | 1 |  | 1 |
| 4 | **Doig GS，2015** | 1 | 1 |  | 1 |  |  | 1 | 1 | 1 |
| 5 | **Jakob SM，2017** |  | 1 |  | 1 |  |  | 1 | 1 | 1 |
| 6 | **Fetterplace K，2018** |  | 1 | 1 | 1 | 1 |  | 1 | 1 | 1 |
| 7 | **van Zanten ARH，2018** |  | 1 | 1 | 1 | 1 |  | 1 | 1 | 1 |
| 8 | **Vega-Alava KM，2018** |  | 1 |  | 1 |  |  | 1 |  | 1 |
| 9 | **de Azevedo JRA，2019** |  | 1 | 1 | 1 | 1 |  | 1 |  | 1 |
| 10 | **Danielis M，2019** |  | 1 |  | 1 | 1 |  | 1 |  | 1 |
| 11 | **Badjatia N，2020** |  | 1 | 1 | 1 | 1 |  |  |  | 1 |
| 12 | **Chapple LS，2020** |  | 1 | 1 | 1 | 1 |  | 1 |  | 1 |
| 13 | **Carteron L，2021** |  | 1 | 1 | 1 | 1 |  | 1 |  | 1 |
| 14 | **Clifton GL，1985** |  | 1 |  | 1 |  |  | 1 |  | 1 |
| 15 | **Mesejo A，2003** |  | 1 |  | 1 |  |  | 1 |  | 1 |
| 16 | **Zhou CP，2006** |  | 1 |  | 1 |  |  |  |  | 1 |
| 17 | **Singer P，2007** |  | 1 | 1 | 1 |  |  | 1 |  | 1 |
| 18 | **Rugeles SJ，2013** |  | 1 | 1 | 1 | 1 |  | 1 |  | 1 |
| 19 | **Ferrie S，2016** | 1 | 1 |  | 1 |  |  | 1 | 1 | 1 |
| 20 | **Heyland DK，2023** |  |  |  | 1 | 1 | 1 | 1 |  | 1 |
| 21 | **Kagan I，2022** |  |  |  | 1 |  |  |  |  | 0 |
| 22 | **Saffle JR，1990** |  |  |  | 1 |  |  | 1 |  | 1 |
| 23 | **de Azevedo JRA，2021** |  |  | 1 | 1 |  | 1 |  |  | 1 |
| 24 | **Bels JLM，2024** |  |  |  |  |  |  | 1 |  | 0 |
| 25 | **Cui-ping Z，2006** |  |  |  |  |  |  | 1 |  | 0 |
| 26 | **Wang Y，2024** |  |  |  |  |  |  | 1 |  | 0 |
| 27 | **Braunschweig CA，2015** | 1 |  |  |  |  |  |  |  | 0 |
| 28 | **Ibrahim EH，2002** | 1 |  |  |  |  |  |  |  | 0 |
| 29 | **Rice TW，2011** | 1 |  |  |  |  |  |  |  | 0 |
| 30 | **Singer P，2011** | 1 |  |  |  |  |  |  |  | 0 |
| 31 | **Hsu CW，2009** | 1 |  |  |  |  |  |  |  | 0 |
| 32 | **Huang HH，2012** | 1 |  |  |  |  |  |  |  | 0 |
| 33 | **Kearns PJ，2000** | 1 |  |  |  |  |  |  |  | 0 |
| 34 | **Goeters C，2002** | 1 |  |  |  |  |  |  |  | 0 |
| 35 | **Heyland D,2013** | 1 |  |  |  |  |  |  |  | 0 |
| 36 | **Ozgultekin A，2008** | 1 |  |  |  |  |  |  |  | 0 |
| 37 | **Qiu C，2015** | 1 |  |  |  |  |  |  |  | 0 |
| 38 | **Xiong W 2021** |  |  | 1 |  | 1 |  |  |  | 1 |
| 39 | **Yeh DD，2020** |  |  | 1 |  | 1 |  |  |  | 1 |
| 40 | **Yeh DD，2017** |  |  | 1 |  |  |  |  |  | 0 |
| 41 | **Zhang Q，2022** |  |  | 1 |  | 1 |  |  |  | 1 |
| 42 | **Lin J,2022** |  |  | 1 |  |  |  |  |  | 0 |
| 43 | **Allingstrup MJ，2017** |  |  | 1 |  | 1 |  |  |  | 1 |
| 44 | **Ridley, E.J，2018** |  |  |  |  |  |  |  | 1 | 0 |
| 45 | **Berger, M.M，2019** |  |  |  |  |  |  |  | 1 | 0 |
| 46 | **Beale, R.J，2008** |  |  |  |  |  |  |  | 1 | 0 |
| 47 | **Tuncay, P，2018** |  |  |  |  |  |  |  | 1 | 0 |
| 48 | **Reilly, J，1990** |  |  |  |  |  |  |  | 1 | 0 |
| 49 | **Wischmeyer, P.E，2017** |  |  |  |  |  |  |  | 1 | 0 |
| 50 | **Zhu, R，2018** |  |  |  |  |  |  |  | 1 | 0 |
| 51 | **Nakamura K，2020** |  |  |  |  |  |  |  | 1 | 0 |
| **Total** | | 13 | 19 | 17 | 23 | 15 | 3 | 22 | 14 | 26 |
| **% Overlapping** | | 50.98% | | | | | | | | |
| **Covered Area(CA)** | | 30.88% | | | | | | | | |
| **Corrected Covered Area(CCA)** | | 29.50% | | | | | | | | |

**additional file B1(Figures)**

**Fig. 1 Risk of bias summary of ROB 2.0**


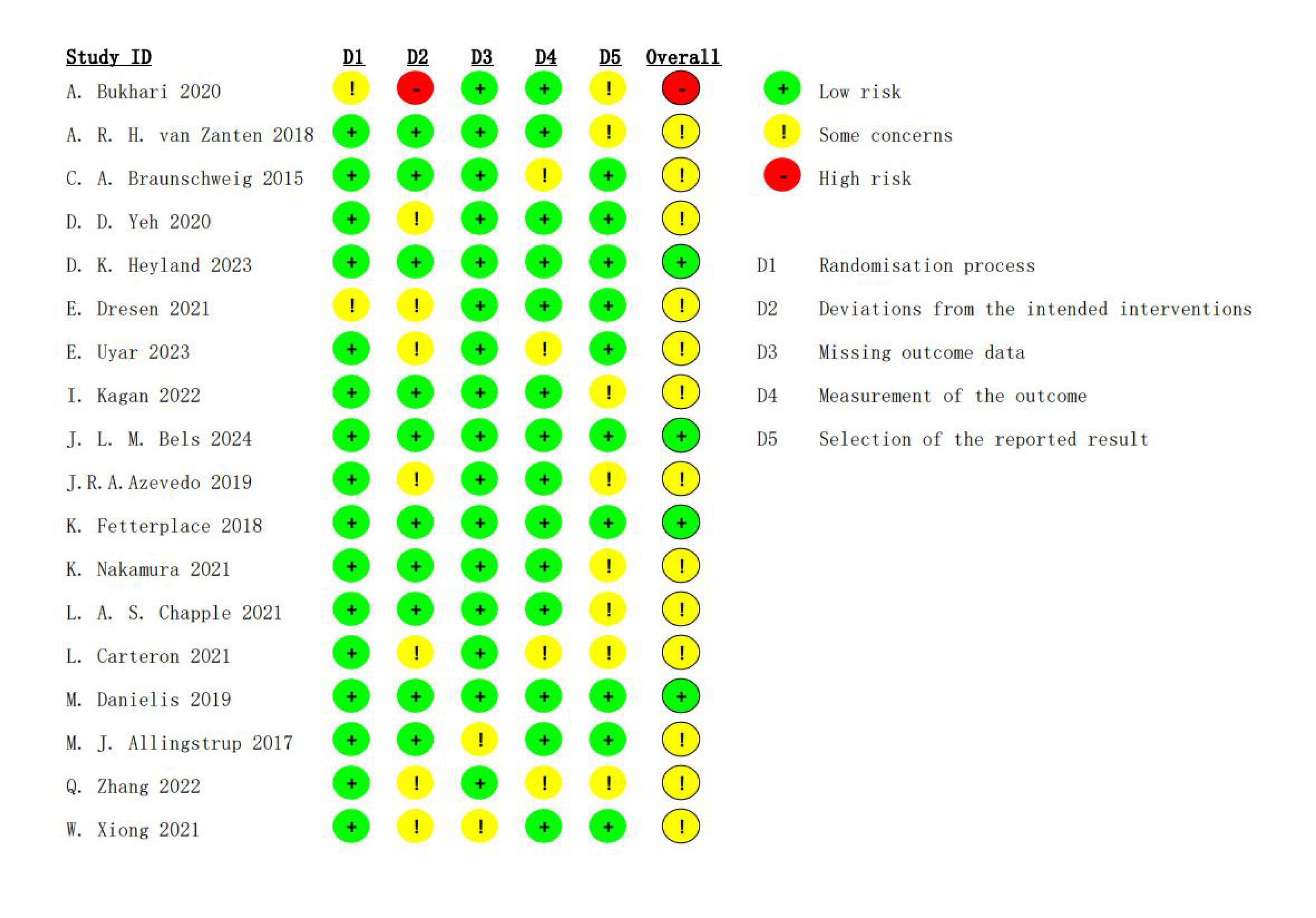


**Fig. 2 The subgroup analysis of overall mortality**


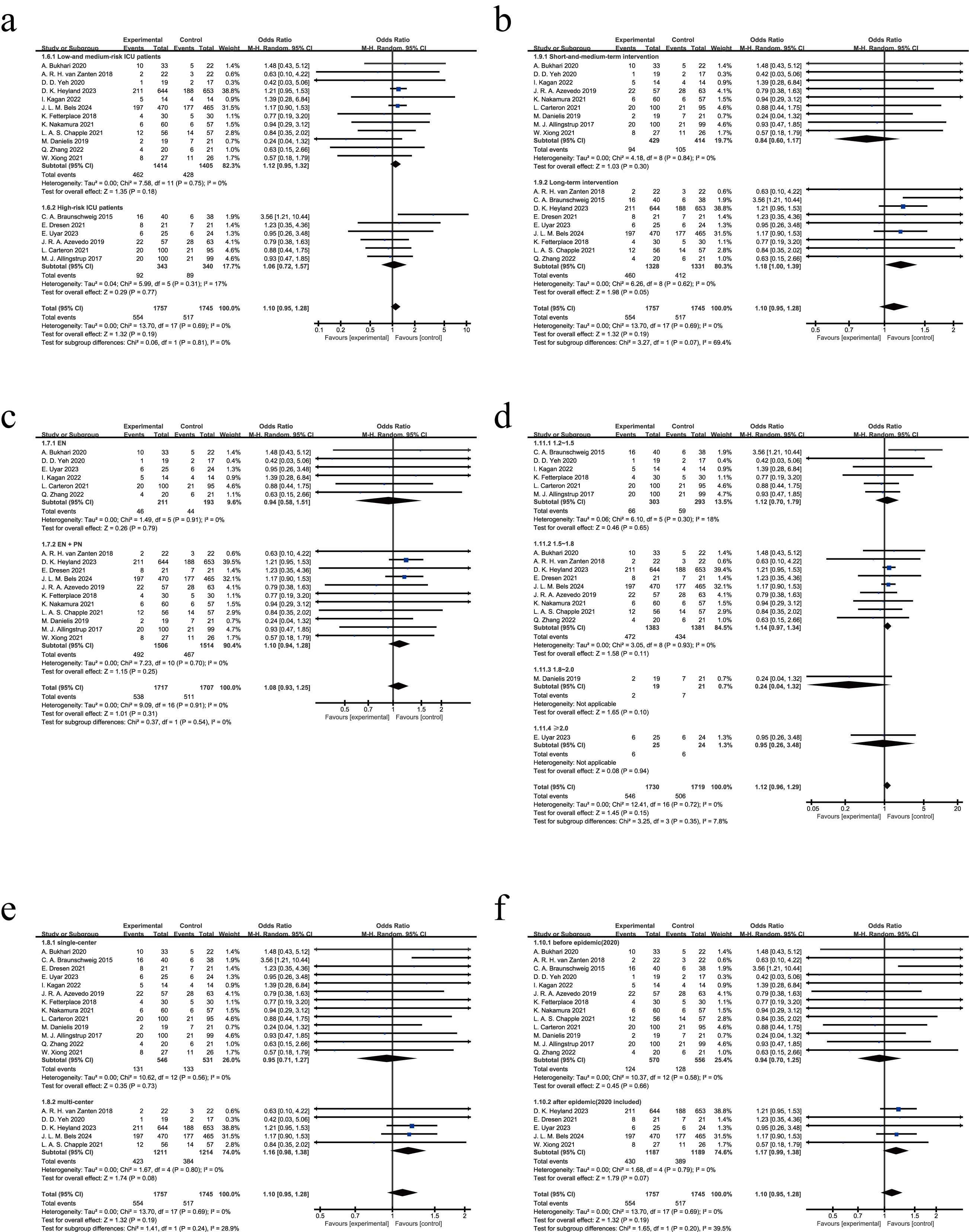


**a.** Low- and medium-risk patients VS high-risk patients, **b.** short- and medium-term intervention VS long term intervention, **c.** EN VS EN+PN, **d.** protein dosage(g/kg/d): 1.2-1.5 VS 1.5-1.8 VS 1.8-2.0 VS ≥2.0, **e.** single VS multiple-center, **f.** before the epidemic(2020) VS after the epidemic(2020 included)

**Fig. 3** **Secondary outcomes**


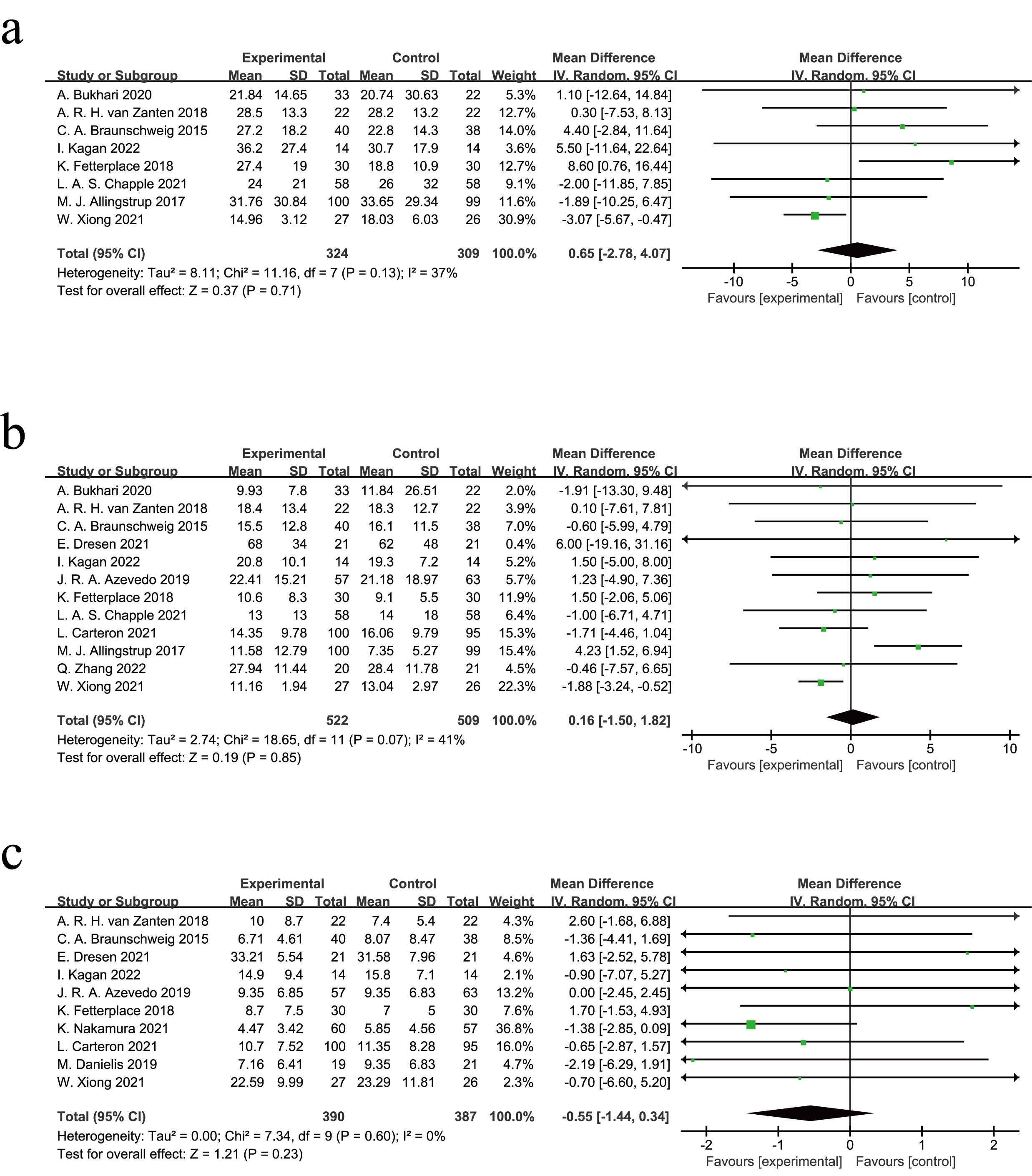


**a.** Lenth of hospital stay**, b.** Lenth of ICU stay, **c.** Mechanical ventilation time

**additional file C(Others)**

**additional file C1**

**Key formula**

1. ​**​ Overall Mean: ​**​$M= \frac{\sum_{i=1}^{k} (n_{i}\times\bar{x_{i}})}{\sum_{i=1}^{k} n_{i}}$
   - *M* = overall mean
   - $n_{i}$= sample size of group i
   - $\bar{x_{i}}$​ = mean of group i
2. ​**​ Within-Group Sum of Squares :​**​$SSW= \sum_{i=1}^{k} \left( n_{i} -1 \right)\times s_{i}^{2}$
   - *SSW* = sum of squares within groups
   - $s_{i}^{2}$​ = variance of group i
3. ​**​ Between-Group Sum of Squares :​**​$SSB= \sum_{i=1}^{k} n_{i}\times{(\bar{x_{i}} -M)}^{2}$
   - *SSB* = sum of squares between groups
   - *M* = overall mean
4. ​**​ Pooled Variance :​**​$s_{p}^{2}= \frac{SSW + SSB}{N -1}$
   - $s_{p}^{2}$​ = pooled variance
   - *N* = total sample size
5. ​**​ Pooled Standard Deviation :​**​$s_{p}= \sqrt{s_{p}^{2}}$

Calculation Process:

1. ​​Input​​ the sample size, mean, and standard deviation for both High-protein polymeric and Oligomeric groups under TBI and Non-TBI conditions.
2. ​​Calculate​​ the total sample size $N=\sum n_{i}$.
3. ​​Calculate​​ the weighted overall mean M.
4. ​​Compute​​ the within-group variation (SSW) and between-group variation (SSB) separately.
5. ​​Calculate​​ the pooled variance using $\frac{SSW + SSB}{N -1}$​.
6. ​​Take the square root​​ to obtain the pooled standard deviation.
7. ​​Output​​ the pooled mean and standard deviation.

**additional file C2**

Python code

**Control group**

def input_control_subgroup(subgroup_name):

"""Input data for a control subgroup"""

n = int(input(f"Enter sample size for 【Control group-{subgroup_name}】 (e.g., enter 5): "))

mean = float(input(f"Enter mean for 【Control group-{subgroup_name}】 (e.g., enter 35.33): "))

sd = float(input(f"Enter standard deviation for 【Control group-{subgroup_name}】 (e.g., enter 52.58): "))

return n, mean, sd

def calculate_control_total(n_values, mean_values, sd_values):

"""Calculate combined mean and standard deviation for control group"""

total_n = sum(n_values)

total_mean = sum(n * m for n, m in zip(n_values, mean_values)) / total_n

# Calculate combined variance

sum_within = sum((n-1) * (sd**2) for n, sd in zip(n_values, sd_values))

sum_between = sum(n * (m - total_mean)**2 for n, m in zip(n_values, mean_values))

total_var = (sum_within + sum_between) / (total_n - 1)

return total_mean, total_var**0.5

# Interactive input for control group data

print("=== Control Group Data Input ===")

control_groups = [

input_control_subgroup("TBI"),

input_control_subgroup("Non-TBI")

]

# Extract data

n_vals = [g[0] for g in control_groups]

mean_vals = [g[1] for g in control_groups]

sd_vals = [g[2] for g in control_groups]

# Calculate results

merged_mean, merged_sd = calculate_control_total(n_vals, mean_vals, sd_vals)

# Output results

print("\n=== Control Group Combined Results ===")

print(f"Total sample size: {sum(n_vals)}")

print(f"Combined mean ± SD: {merged_mean:.2f} ± {merged_sd:.2f}")

**Intervention group**

def input_group_data(group_name, subgroup_name):

"""Input data for a single subgroup"""

n = int(input(f"Enter sample size for 【{group_name}-{subgroup_name}】: "))

mean = float(input(f"Enter mean value for 【{group_name}-{subgroup_name}】: "))

sd = float(input(f"Enter standard deviation for 【{group_name}-{subgroup_name}】: "))

return n, mean, sd

def calculate_total(n_values, mean_values, sd_values):

"""Calculate combined mean and standard deviation"""

total_n = sum(n_values)

total_mean = sum(n * mean for n, mean in zip(n_values, mean_values)) / total_n

# Calculate combined variance

sum_within_variance = sum((n - 1) * (sd ** 2) for n, sd in zip(n_values, sd_values))

sum_between_variance = sum(n * ((mean - total_mean) ** 2) for n, mean in zip(n_values, mean_values))

total_variance = (sum_within_variance + sum_between_variance) / (total_n - 1)

total_sd = total_variance ** 0.5

return total_mean, total_sd

# Interactive data input

print("=== Enter group data ===")

groups = []

# Input Oligomeric protein groups

groups.append(input_group_data("Oligomeric", "TBI"))

groups.append(input_group_data("Oligomeric", "Non-TBI"))

# Input High-protein polymeric groups

groups.append(input_group_data("High-protein polymeric", "TBI"))

groups.append(input_group_data("High-protein polymeric", "Non-TBI"))

# Extract data

n_values = [group[0] for group in groups]

mean_values = [group[1] for group in groups]

sd_values = [group[2] for group in groups]

# Calculate results

total_mean, total_sd = calculate_total(n_values, mean_values, sd_values)

# Output results

print("\n=== Combined Results ===")

print(f"Total sample size: {sum(n_values)}")

print(f"Combined mean ± SD: {total_mean:.2f} ± {total_sd:.2f}")
