# Supplementary material for: High-protein supplementation in critically ill patients: a systematic review, meta-analysis and umbrella review of existing evidence
Source: Front Nutr. 2026 May 21;13:1788894. doi: 10.3389/fnut.2026.1788894 (PMC13233266; doi:10.3389/fnut.2026.1788894)
Supplement: Supplementary file 2 [file Table_2.DOCX]

**Additional file 2**

**additional file B2(Figures)**

**List of Additional file 2**

[**additional file B2(Figures)** 1](#_Toc204372252)

[**Fig. 4 The subgroup analysis of length of hospital stay** 3](#_Toc204372253)

[**Fig. 5 The subgroup analysis of length of ICU stay** 4](#_Toc204372254)

[**Fig. 6 The subgroup analysis of mechanical ventilation time** 5](#_Toc204372255)

[**Fig. 7 Adverse event outcomes** 6](#_Toc204372256)

[**Fig. 8 Other outcomes of the umbrella review** 7](#_Toc204372257)

**Fig. 4 The subgroup analysis of length of hospital stay**


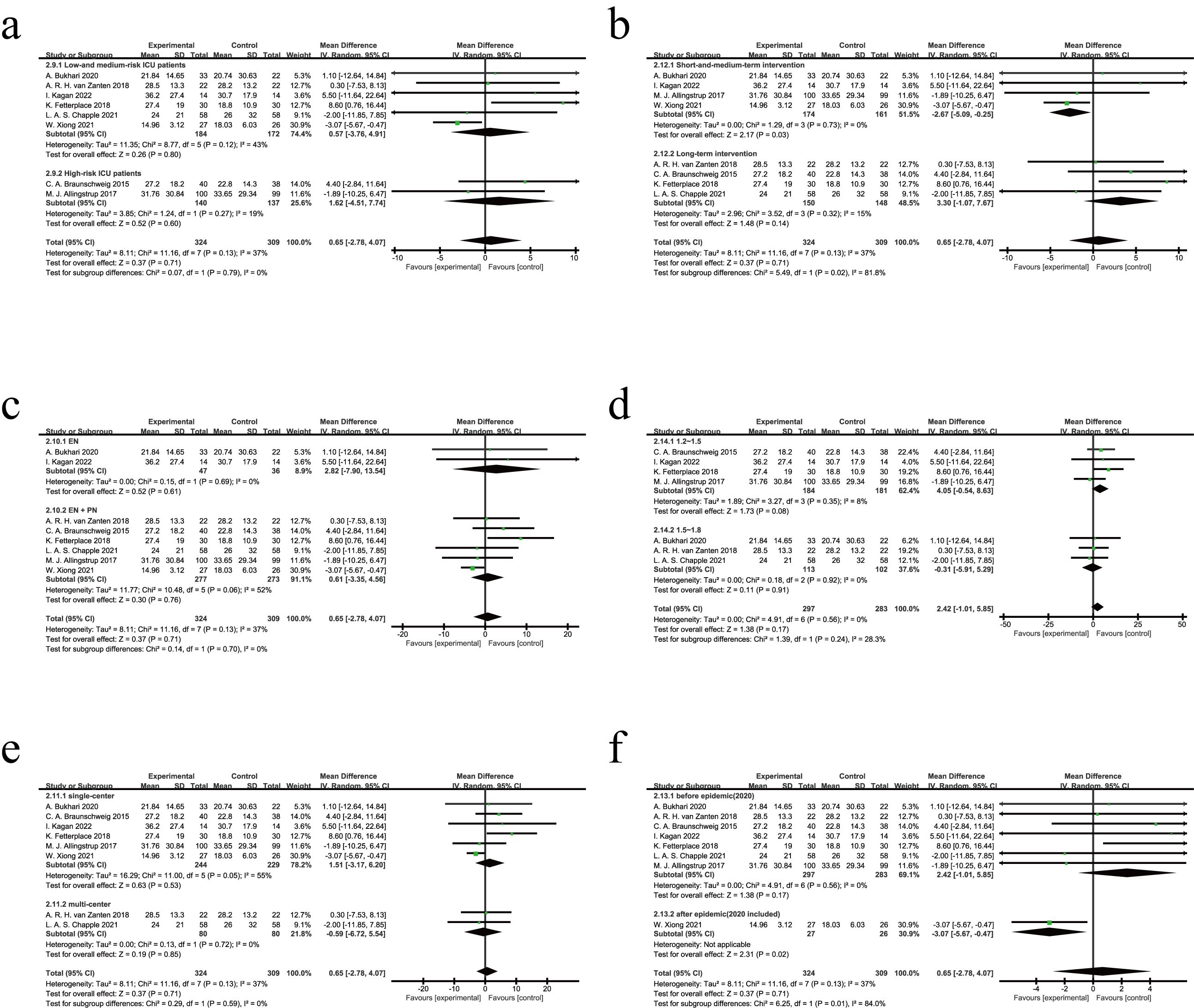


**a.** Low- and medium-risk patients VS high-risk patients, **b.** short- and medium-term intervention VS long term intervention, **c.** EN VS EN+PN, **d.** protein dosage(g/kg/d): 1.2-1.5 VS 1.5-1.8 VS 1.8-2.0 VS ≥2.0, **e.** single VS multiple-center, **f.** before the epidemic(2020) VS after the epidemic(2020 included)

**Fig. 5 The subgroup analysis of length of ICU stay**


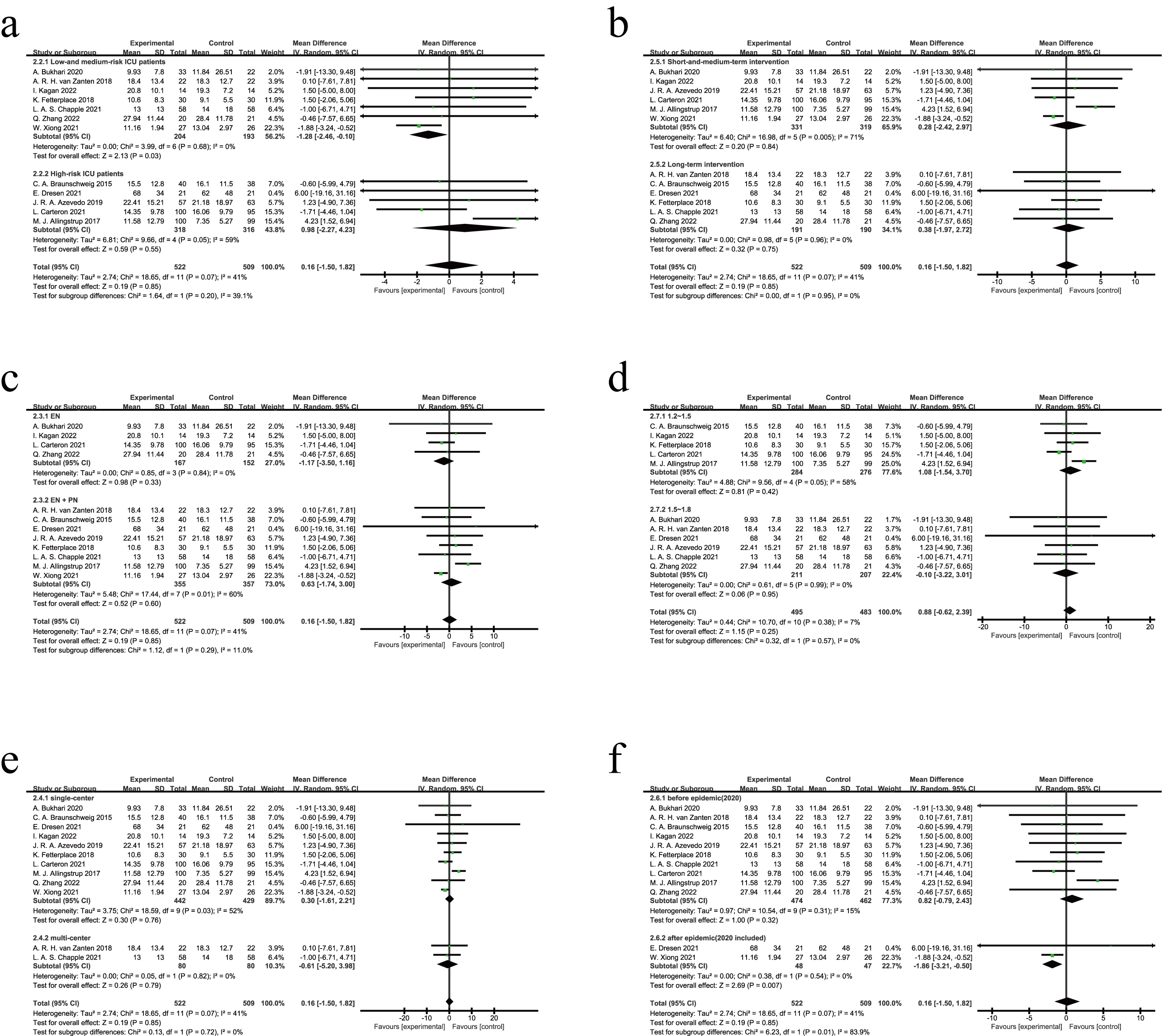


**a.** Low- and medium-risk patients VS high-risk patients, **b.** short- and medium-term intervention VS long term intervention, **c.** EN VS EN+PN, **d.** protein dosage(g/kg/d): 1.2-1.5 VS 1.5-1.8 VS 1.8-2.0 VS ≥2.0, **e.** single VS multiple-center, **f.** before the epidemic(2020) VS after the epidemic(2020 included)

**Fig. 6 The subgroup analysis of mechanical ventilation time**


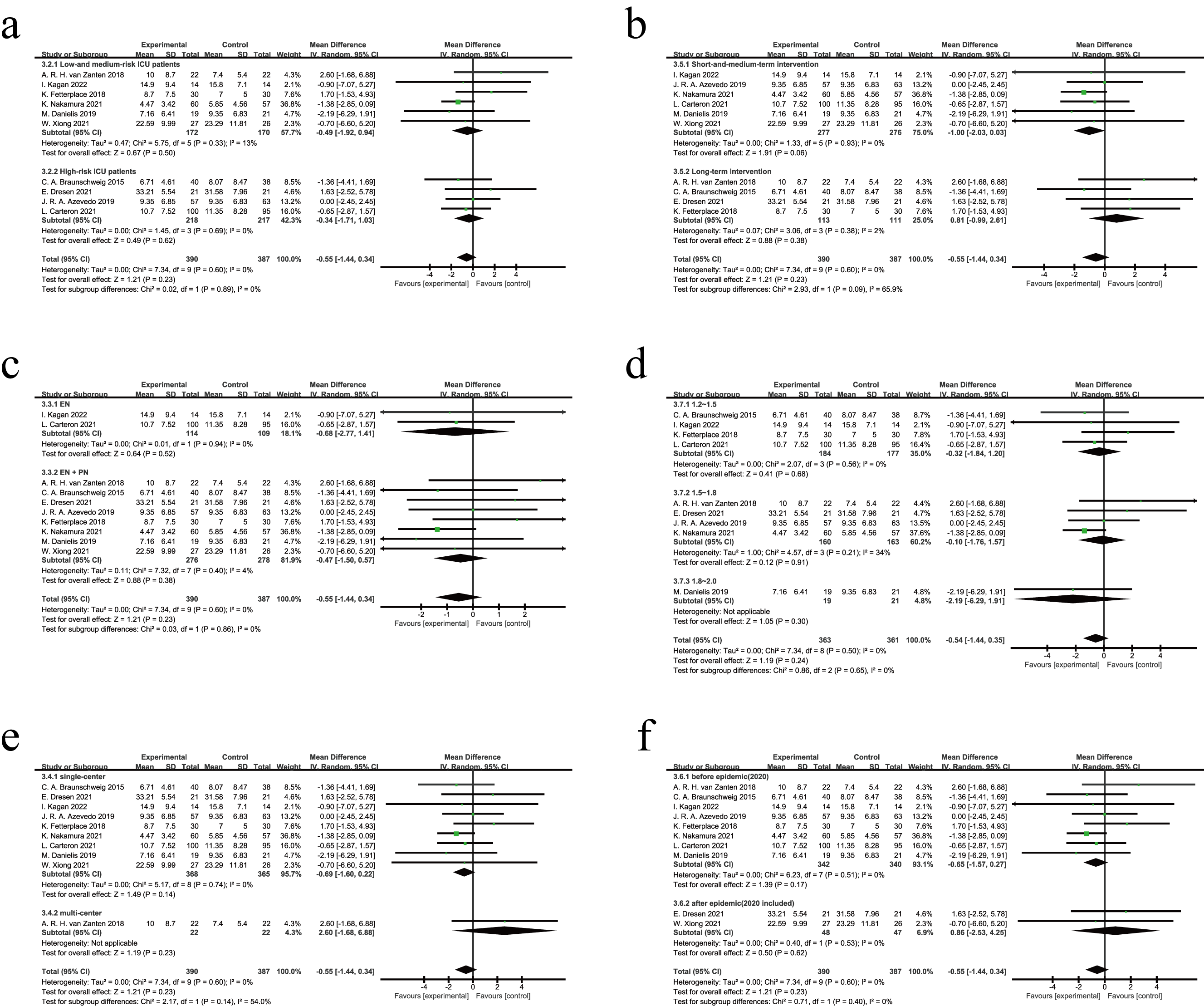


**a.** Low- and medium-risk patients VS high-risk patients, **b.** short- and medium-term intervention VS long term intervention, **c.** EN VS EN+PN, **d.** protein dosage(g/kg/d): 1.2-1.5 VS 1.5-1.8 VS 1.8-2.0 VS ≥2.0, **e.** single VS multiple-center, **f.** before the epidemic(2020) VS after the epidemic(2020 included)

**Fig. 7 Adverse event outcomes**


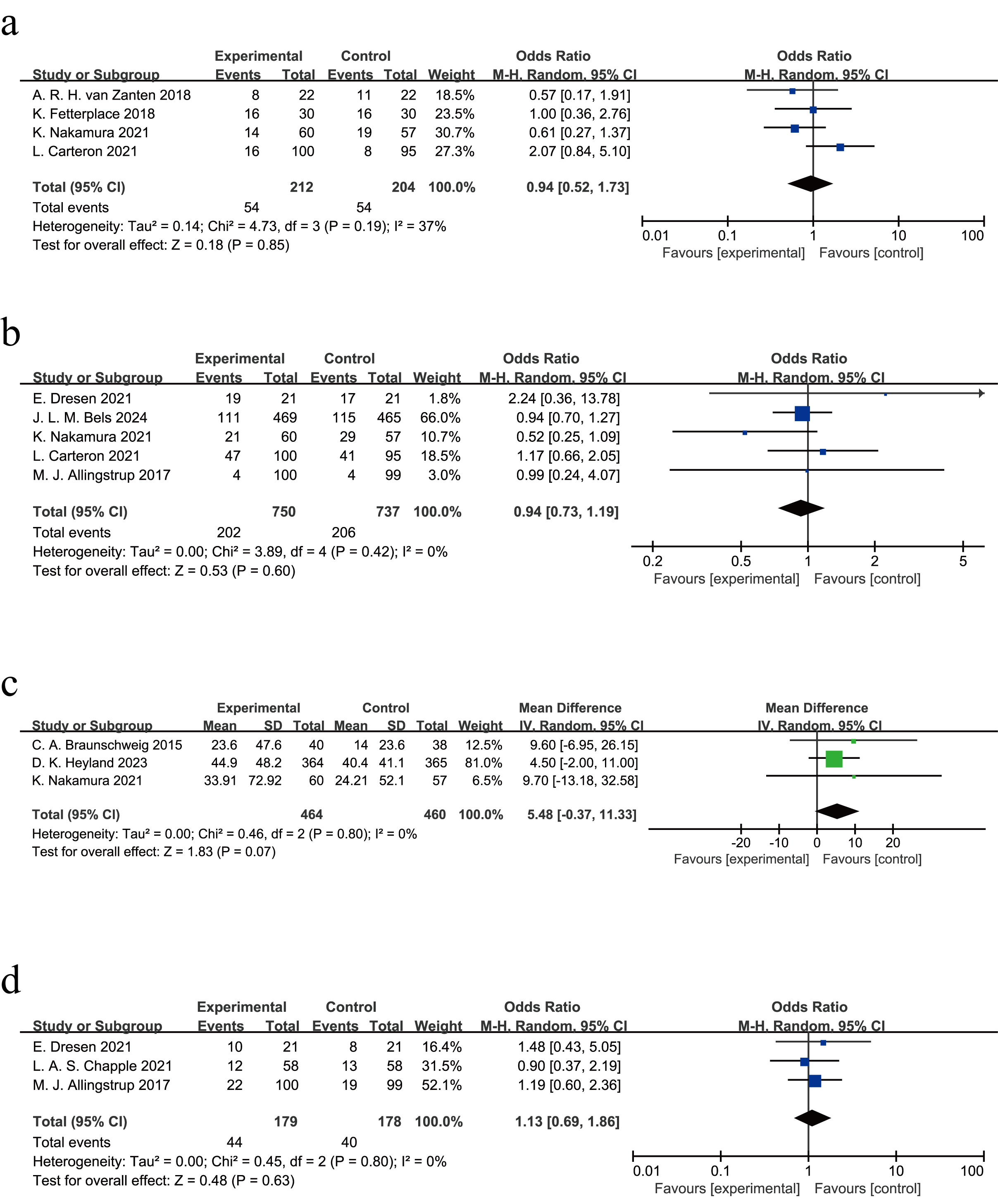


**a.** Diarrhea rate, **b.** Pneumonia infection rate, **c.** Insulin requirement(IU), **d.** RRT rate

**Fig. 8** **Other outcomes of the umbrella review**


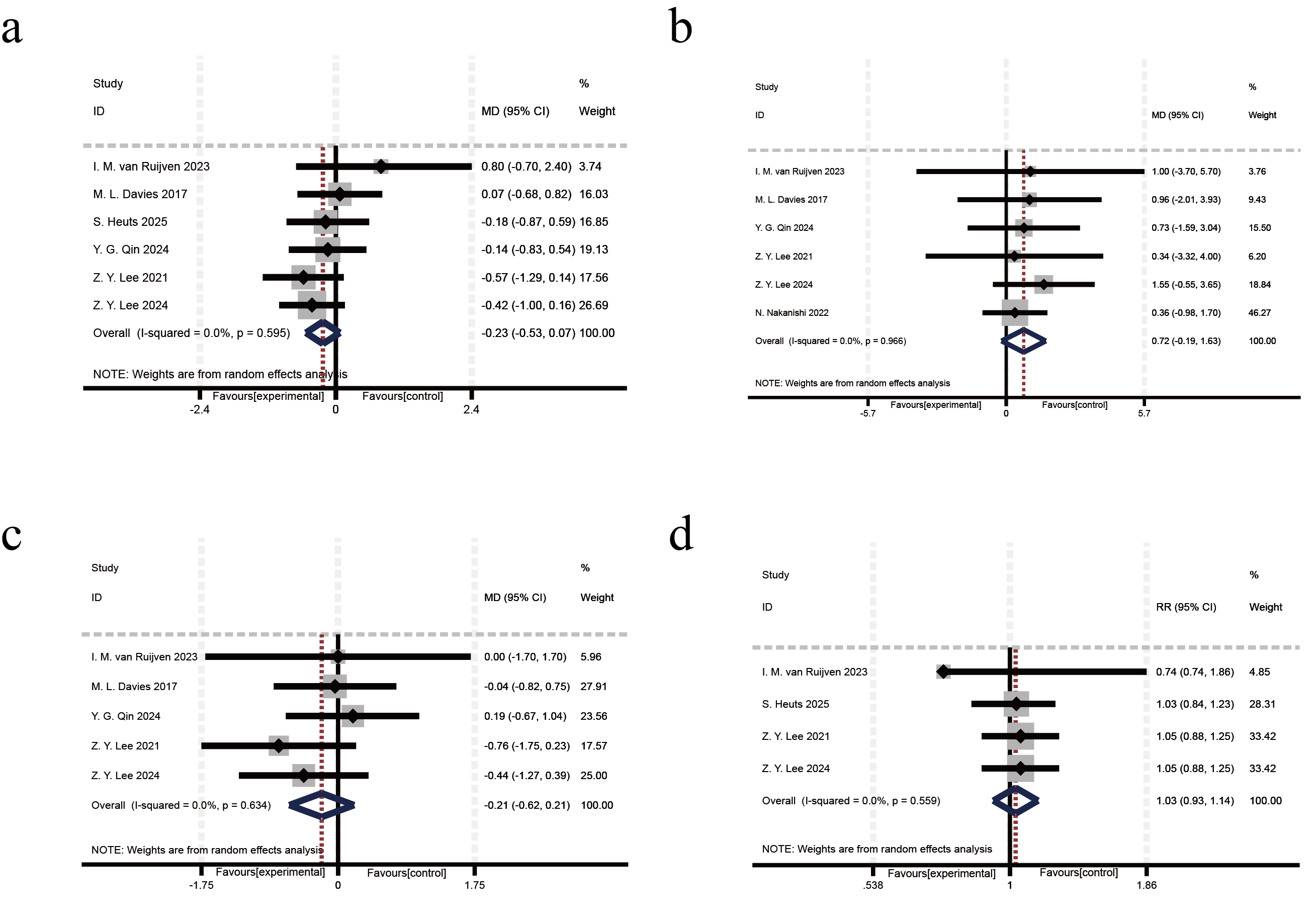


**a.** Mechanical ventilation time(day), **b.** Length of hospital stay(day), **c.** Length of ICU stay(day), **d.** Infectious complication rate
