# Supplementary material for: High-protein supplementation in critically ill patients: a systematic review, meta-analysis and umbrella review of existing evidence
Source: Front Nutr. 2026 May 21;13:1788894. doi: 10.3389/fnut.2026.1788894 (PMC13233266; doi:10.3389/fnut.2026.1788894)
Supplement: Supplementary file 3 [file Table_3.DOCX]

**Additional file 3**

**additional file B3(Figures)**

**List of Additional file 3**

[**additional file B3(Figures)** 1](#_Toc204372693)

[**Fig. 9 Trial sequential analysis(TSA)** 3](#_Toc204372694)

[**Fig. 10 Publication bias by funnel plots for overall mortality** 4](#_Toc204372695)

[**Fig. 11 GRADE certainty assessments** 5](#_Toc204372696)

[**Fig. 12 The graphic digitization function processing in Origin64** 6](#_Toc204372697)

**Fig. 9 Trial sequential analysis(TSA)**


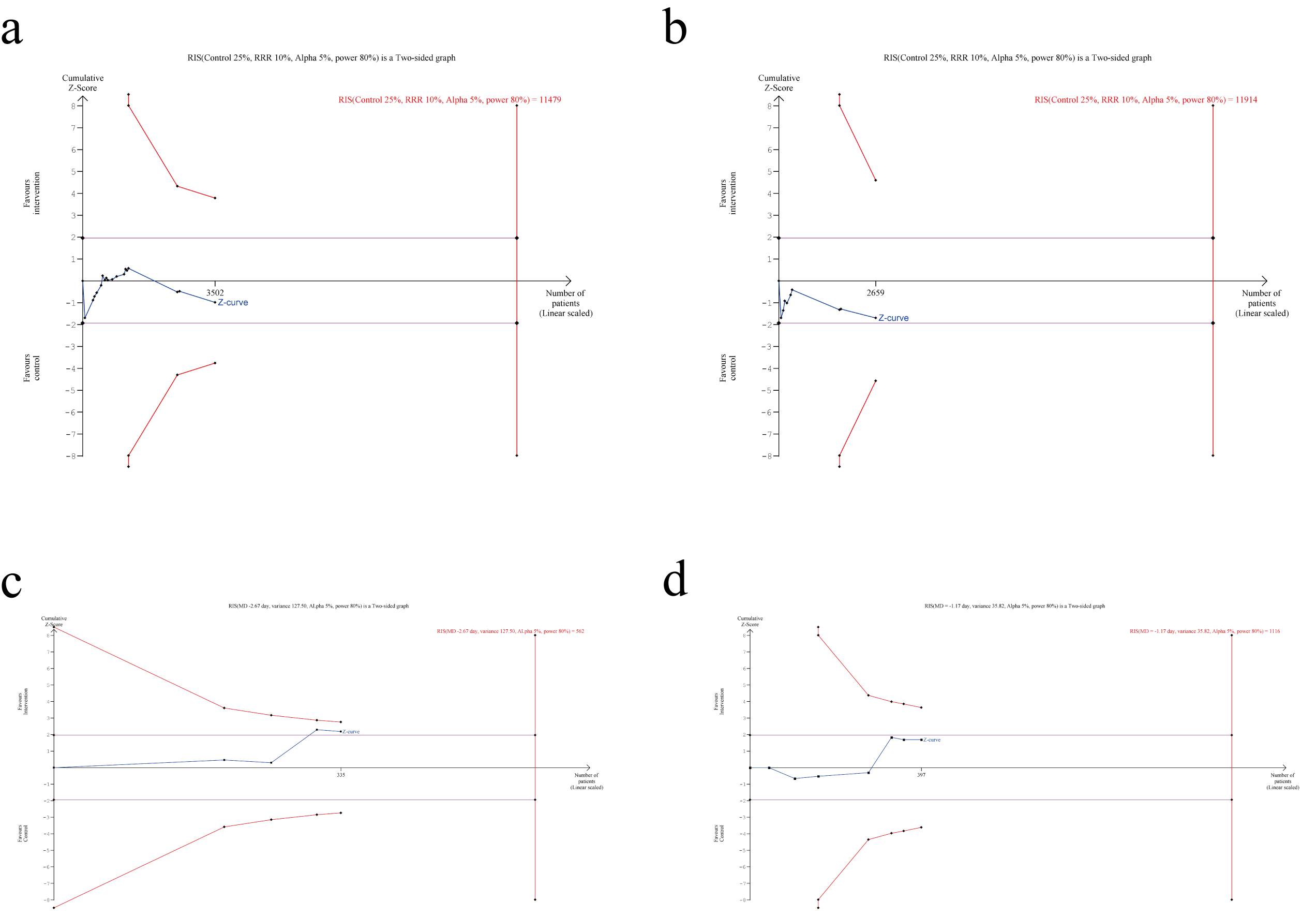


**a.** Overall mortality(18 studies, n = 3502), **b.** Long-term intervention subgroup analysis of overall mortality(9 studies, n = 2659), **c.** Short- and medium-term intervention subgroup analysis of length of hospital stay(4 studies, n = 335), **d.** Low- and medium-risk patients subgroup analysis of length of ICU stay(7 studies, n = 397)

TSA was analysed using Biggerstaff-Tweedie(BT) random‑effects model. The Z curve in blue measures the treatment effect (pooled relative risk). The parallel lines in pink are the boundaries of conventional meta‑analysis (Z = 1.96), and the boundaries of benefit and harm are boundaries of conventional meta‑analysis adjusted for between‑trial heterogeneity and multiple statistical testing (TSA boundaries). A treatment effect outside the TSA boundaries(the curve in red) of benefit/harm indicates reliable evidence for a treatment effect. The red vertical line on the far right is RIS. RIS: required information size, MD: mean difference, RRR: relative risk reduction, TSA: trial sequential analysis.

**Fig. 10 Publication bias by funnel plots for overall mortality**

**
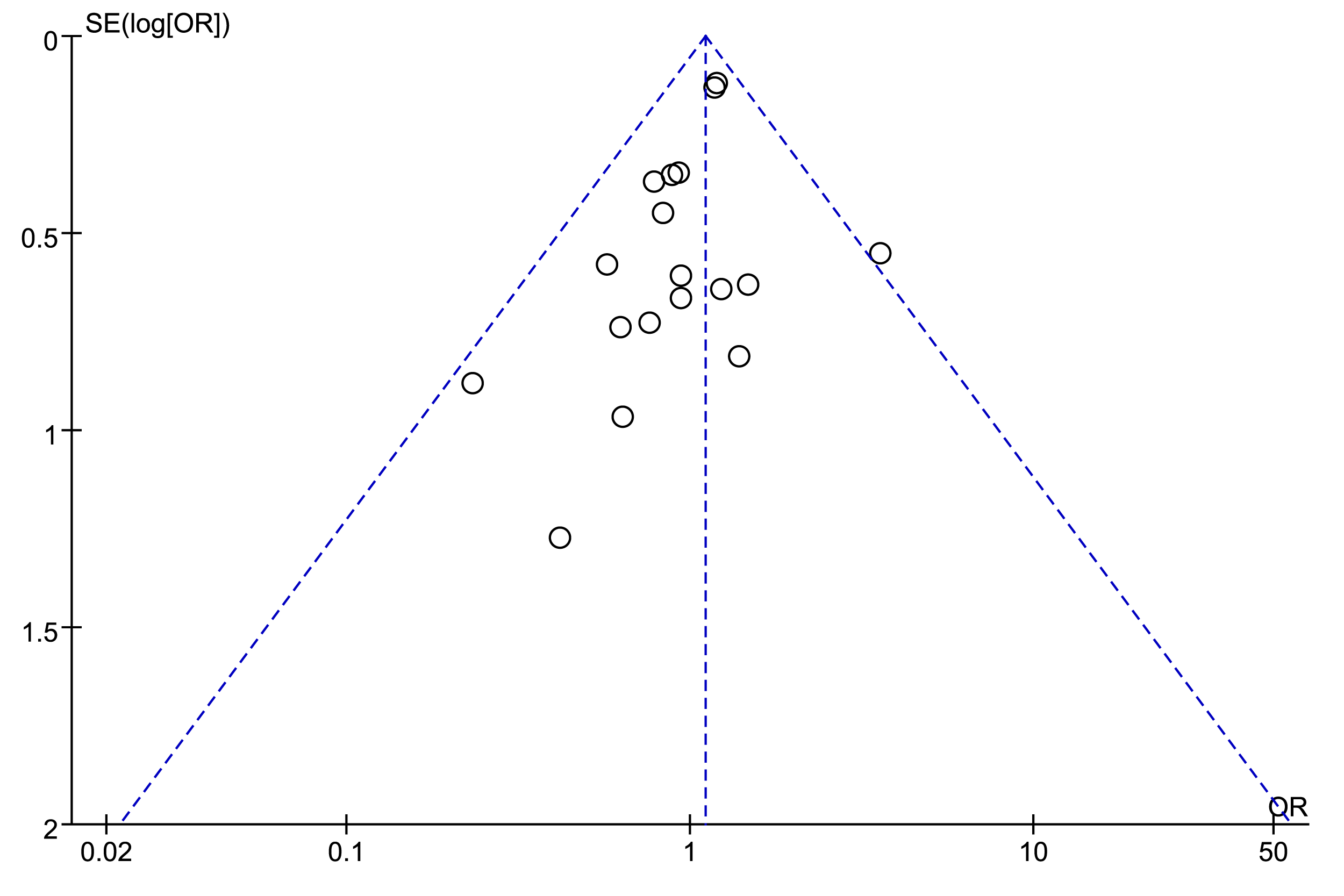
**

meta bias, egger random(dlaird)

Effect-size label: Log odds-ratio

Effect size: _meta_es

Std. err.: _meta_se

Regression-based Egger test for small-study effects

Random-effects model

Method: DerSimonian–Laird

H0: beta1 = 0; no small-study effects

beta1 = -0.59

SE of beta1 = 0.364

z = -1.61

Prob > |z| = 0.1074 > 0.05

|z| = 0.1074 > 0.05, there is no publication bias.

meta bias, begg

Effect-size label: Log odds-ratio

Effect size: _meta_es

Std. err.: _meta_se

Begg's test for small-study effects

Kendall's score = -37.00

SE of score = 26.401

z = -1.44

Prob > |z| = 0.1727 > 0.05

|z| = 0.1727 > 0.05, there is no publication bias.

**Fig. 11 GRADE certainty assessments**

**
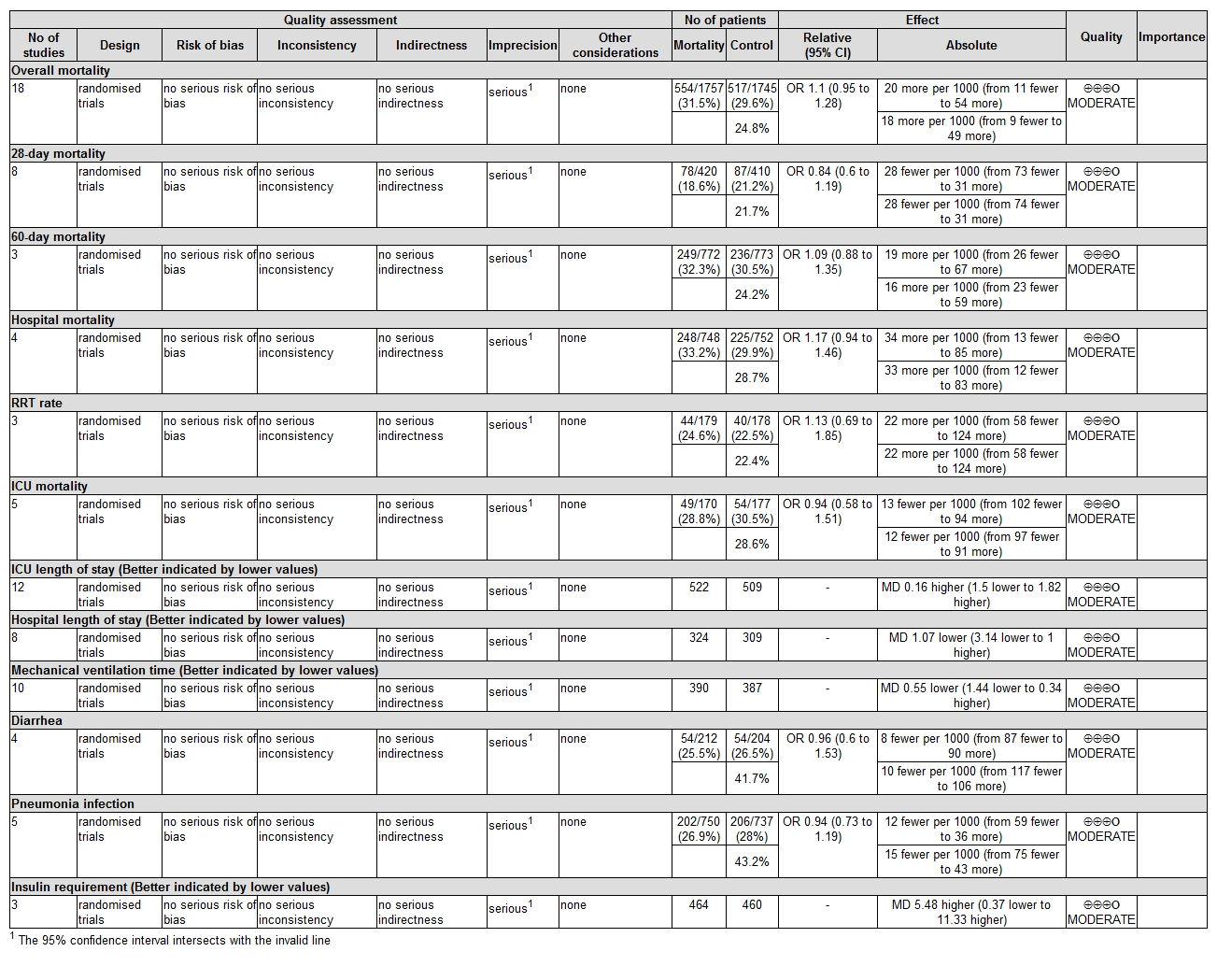
**

**Fig. 12 The graphic digitization function processing in Origin64**


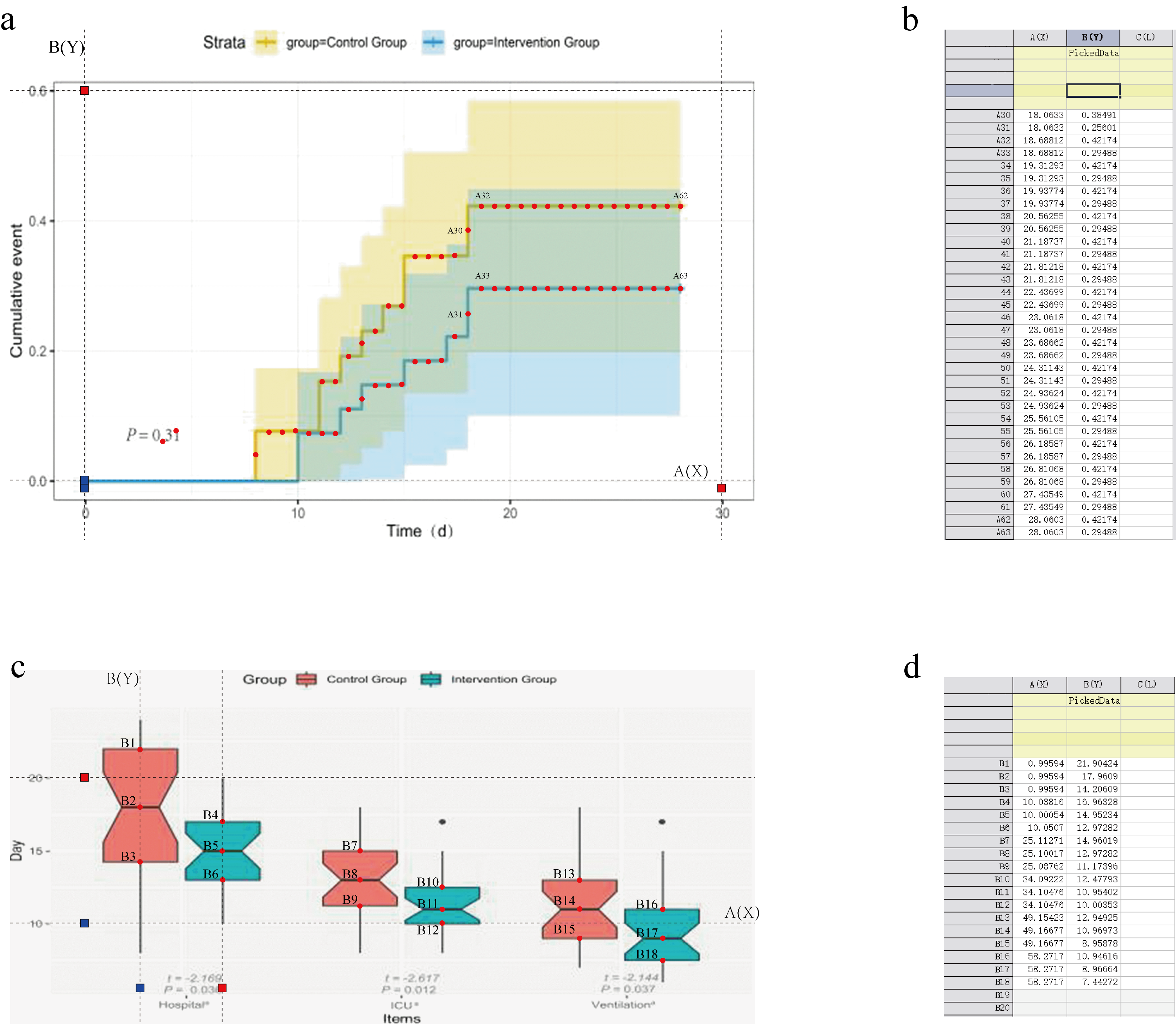


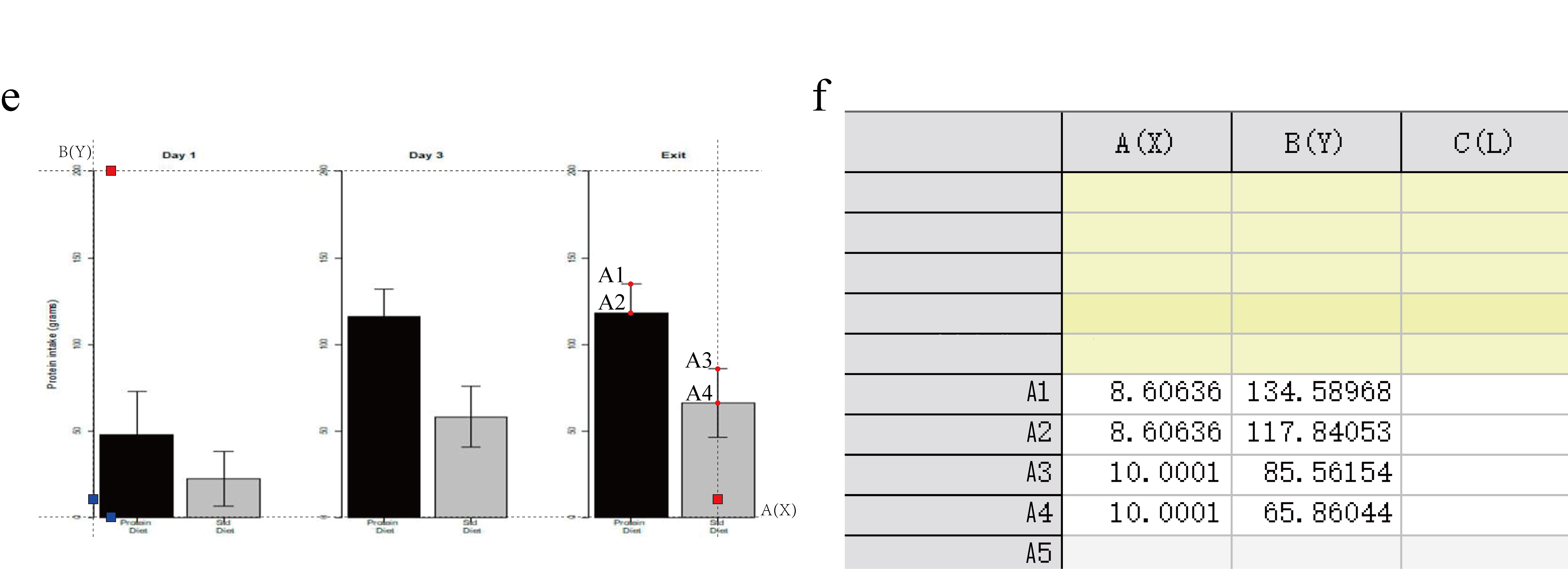


**a.** The 28-day mortality image after tracing with Origin64, **b.** The coordinate values of 28-day mortality after plotting with Origin64, **c.** The images of length of hospital stay, length of ICU stay, and mechanical ventilation time after Origin64 tracing, **d.** The coordinate values of length of hospital stay, length of ICU stay, and mechanical ventilation time after Origin64 tracing, **e.** protein dosage image after Origin64 tracing, **f.** protein dosage coordinate values after tracing with Origin64
