# Supplementary material for: High-protein supplementation in critically ill patients: a systematic review, meta-analysis and umbrella review of existing evidence
Source: Front Nutr. 2026 May 21;13:1788894. doi: 10.3389/fnut.2026.1788894 (PMC13233266; doi:10.3389/fnut.2026.1788894)
Supplement: Supplementary file 4 [file Table_4.DOCX]

**Additional file 4**

**additional file B4(Figures)**

**List of Additional file 4**

[**additional file B4(Figures)** 1](#_Toc226411458)

[**Fig. 13 Sensitivity analysis (Sensitivity meta-analysis excluding three specific types of studies)** 3](#_Toc226411459)

[**Fig. 14 Sensitivity analysis (Sensitivity meta‑analysis restricted to studies reporting direct overall mortality)** 5](#_Toc226411460)

[**Fig. 15 Kappa statistics** 6](#_Toc226411462)

**Fig. 13 Sensitivity analysis (Sensitivity meta-analysis excluding three specific types of studies)**


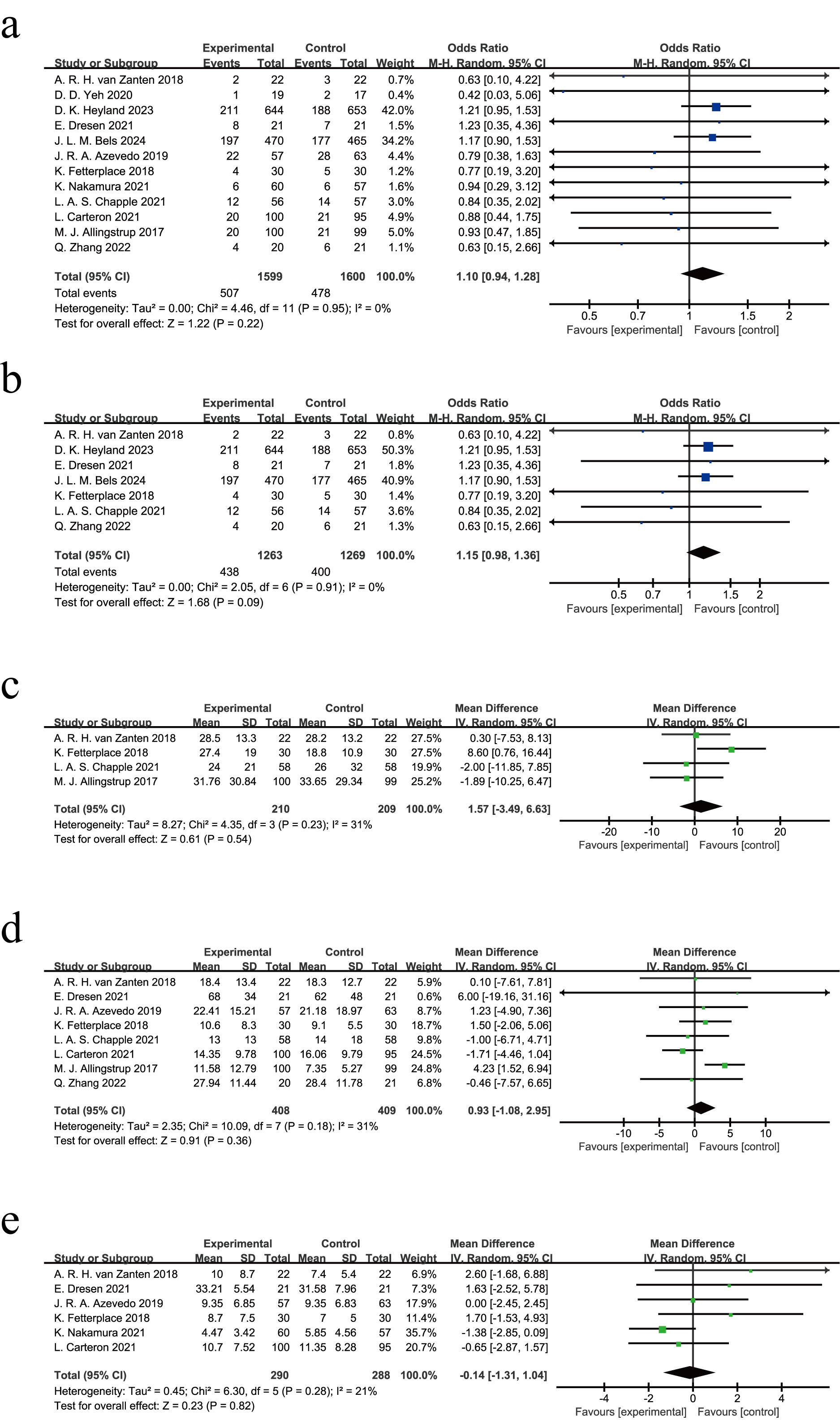


**a.** overall mortality, **b.** the long‑term intervention subgroup of overall mortality, **c.** length of hospital stay, **d.** length of ICU stay, **e.** mechanical ventilation time

**Fig. 14 Sensitivity analysis (Sensitivity meta‑analysis restricted to studies reporting direct overall mortality)**

**
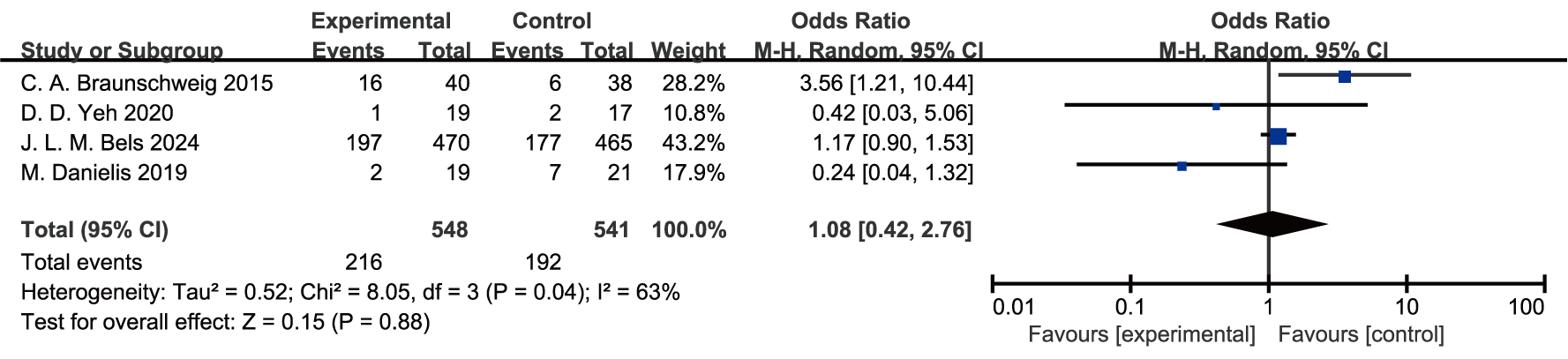
**

**Fig. 15 Kappa statistics**


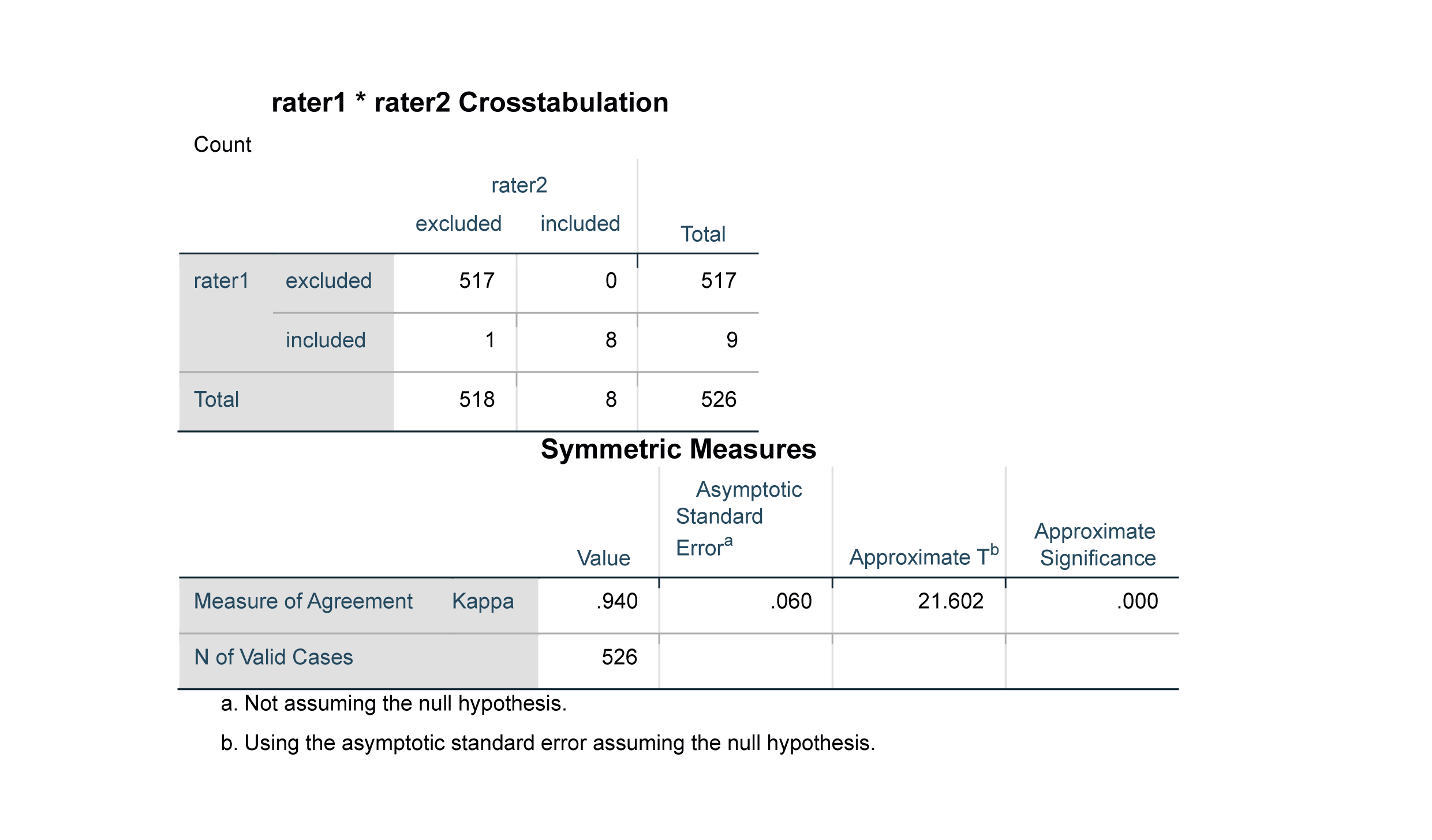


**a.** Kappa statistics of meta-analysis


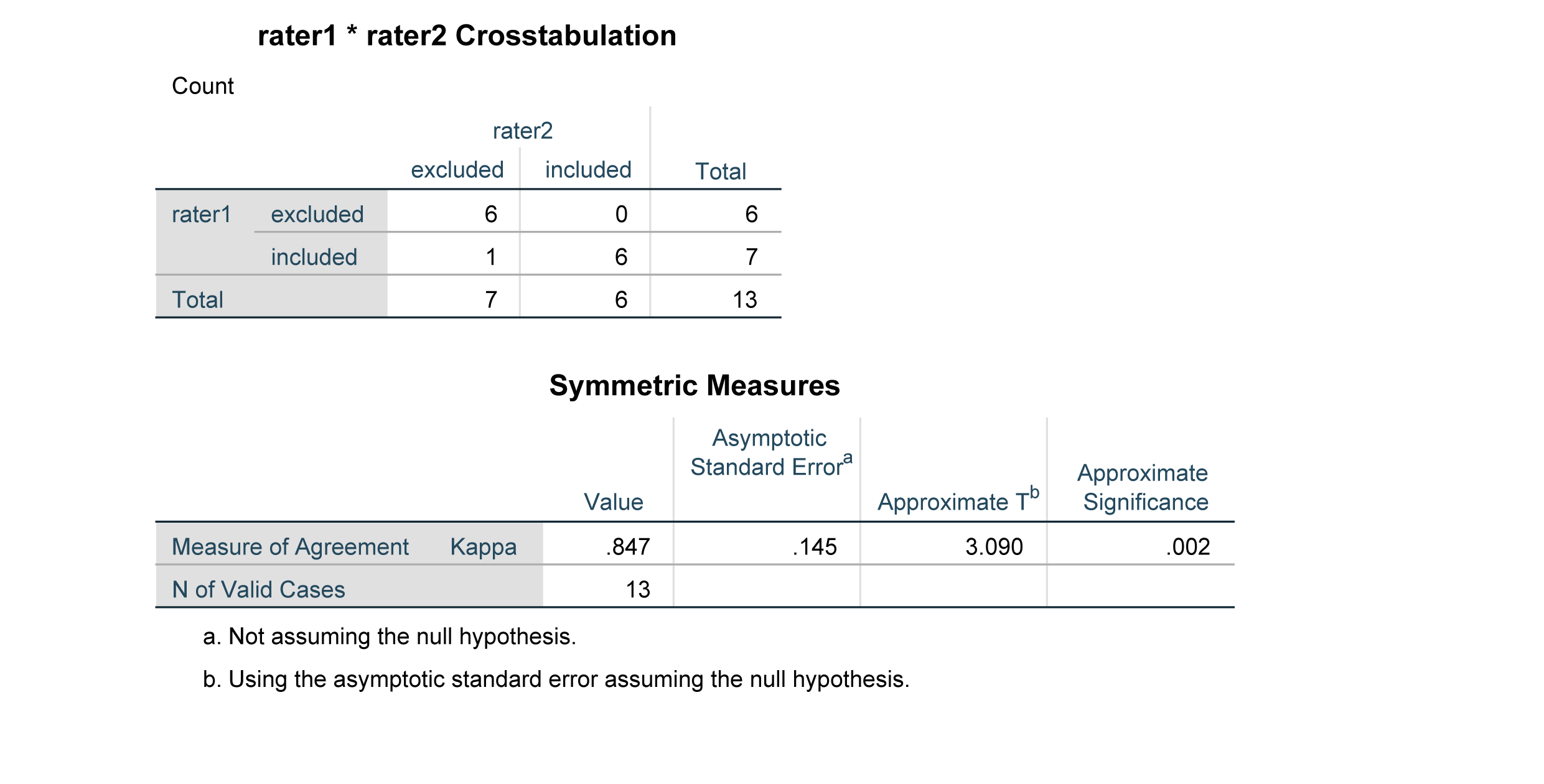


**b.** Kappa statistics of umbrella review
